# Supplementary material for: Systematic discovery of UFM1 receptors reveals a regulatory module in DNA repair directing non-homologous end-joining
Source: Nat Commun. 2026 Jun 15;17:7574. doi: 10.1038/s41467-026-73882-8 (PMC13415529; doi:10.1038/s41467-026-73882-8)
Supplement: Supplementary file 1 — Supplementary Information [file 41467_2026_73882_MOESM1_ESM.pdf]

Supplementary Figure 1

A **Module 1 results - TLR**

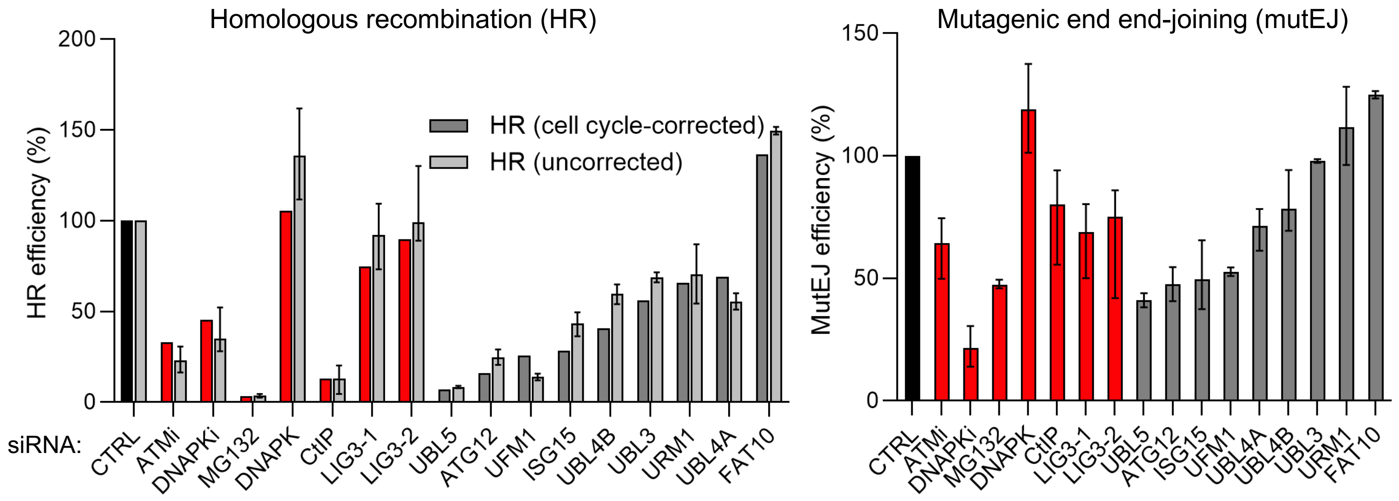

B **Module 2 results – IRIF kinetics**

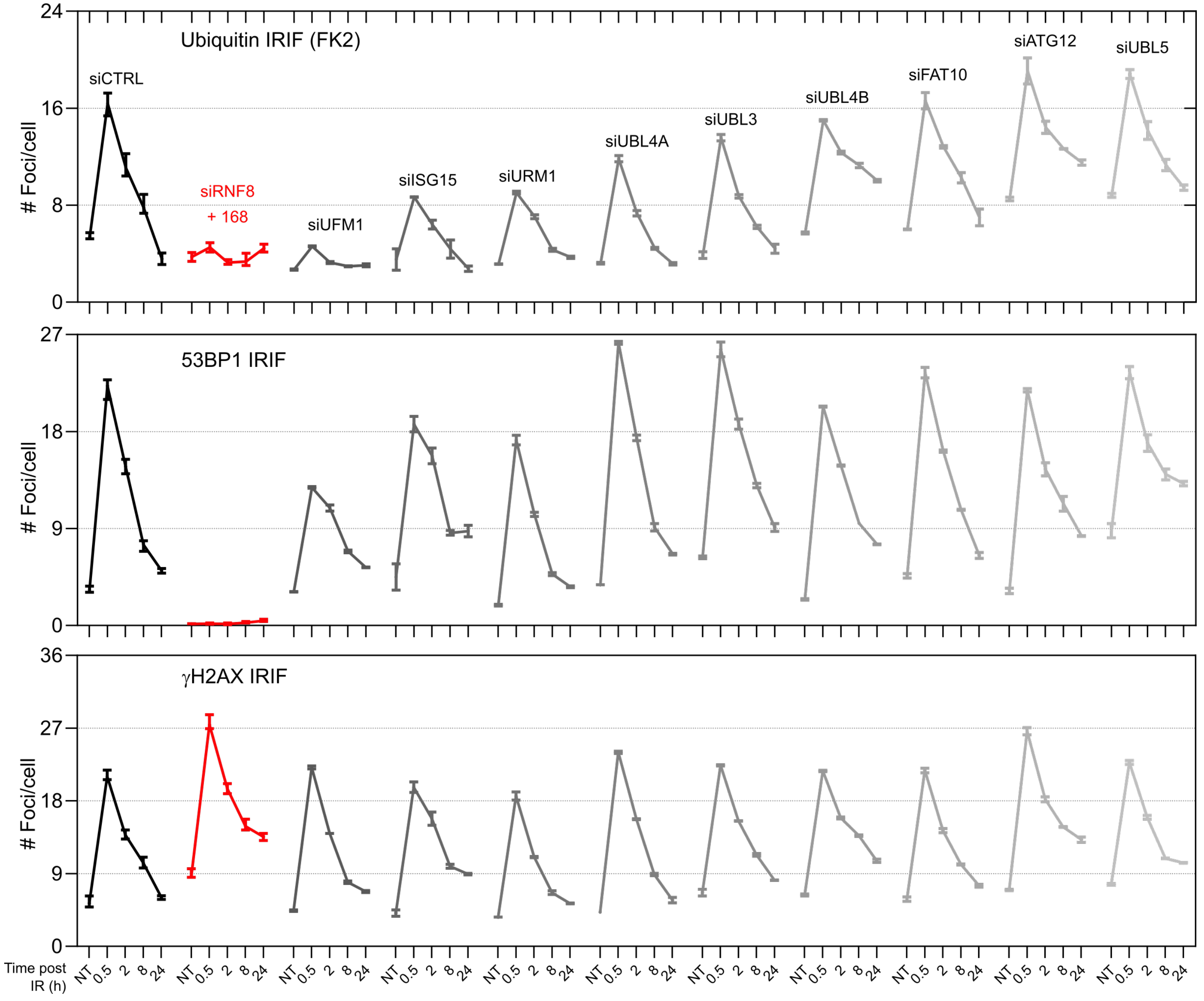

Supplementary Figure 1. **Individual readouts for two-module screening of UBLs for roles in the DNA damage response.** (**A**) Left: Module 1 screening results for homologous recombination (HR) efficiency, using the traffic light reporter (TLR) system integrated into U2OS cells. Given HR's limitation to S/G2, results are presented corrected to flow-cytometry S/G2 values (left bars) in comparison to uncorrected values (right bars). Non-targeting control (CTRL) siRNA targeting luciferase (black) and small molecule inhibitors targeting ATM, DNA-PK and the proteasome (MG132) as well as siRNAs targeting DNAP-PK, CtIP and LIG3 (red) were used as negative and positive controls, respectively. Control conditions were published previously, due to forming part of the same screening pipeline[17]. Data represent means ± ranges of n=9 (siCTRL), n=8 (siCtIP), n=6 (siLIG3-1), n=5 (DNAPKi, siDNA-PK, siLIG3-2), n=4 (ATMi), n=3 (MG132, siISG15, siUBL4A, siUBL4B, siURM1) or n=2 (siUBL5, siATG12, siUFM1, siUBL3, siFAT10) biological replicates. Right: Module 1 screening results for DNA double-strand break repair by mutagenic end-joining (mutEJ), using the traffic light reporter (TLR system) integrated into U2OS cells. Controls are as described in (A). Data represent means ± ranges of n=9 (siCTRL), n=8 (siCtIP), n=6 (siLIG3-1), n=5 (DNAPKi, siDNA-PK, siLIG3-2), n=4 (ATMi), n=3 (MG132, siISG15, siUBL4A, siUBL4B, siURM1) or n=2 (siUBL5, siATG12, siUFM1, siUBL3, siFAT10) biological replicates. (**B**) Recruitment and resolution kinetics of conjugated ubiquitin recognised by the FK2 antibody, 53BP1 and γH2AX ionising radiation-induced foci (IRIF) in U2OS cells at the indicated time points after IR treatment (2 Gy), compared to the non-treated condition. Non-targeting control siRNA (siCTRL, against luciferase) and an siRNA mix targeting RNF8 plus RNF168 were used as negative and positive controls, respectively. Control data were published previously, due to forming part of the same screening pipeline[17]. Plots represent means ± range for n=9 (siCTRL and siRNF8+168) or medians ± range for n=2 (UBL siRNAs) 96-plate wells integrating, on average, >1000 cells imaged per UBL, and >8000 per control siRNA, per condition, with >400,000 cells imaged in total in one screening experiment. Source data for 1A and 1B are provided as a Source Data file.

Abbreviations: CTRL: control; HR: homologous recombination; IRIF: ionising radiation-induced foci; mutEJ: mutagenic end-joining; NT: non-treated; TLR: traffic light reporter.

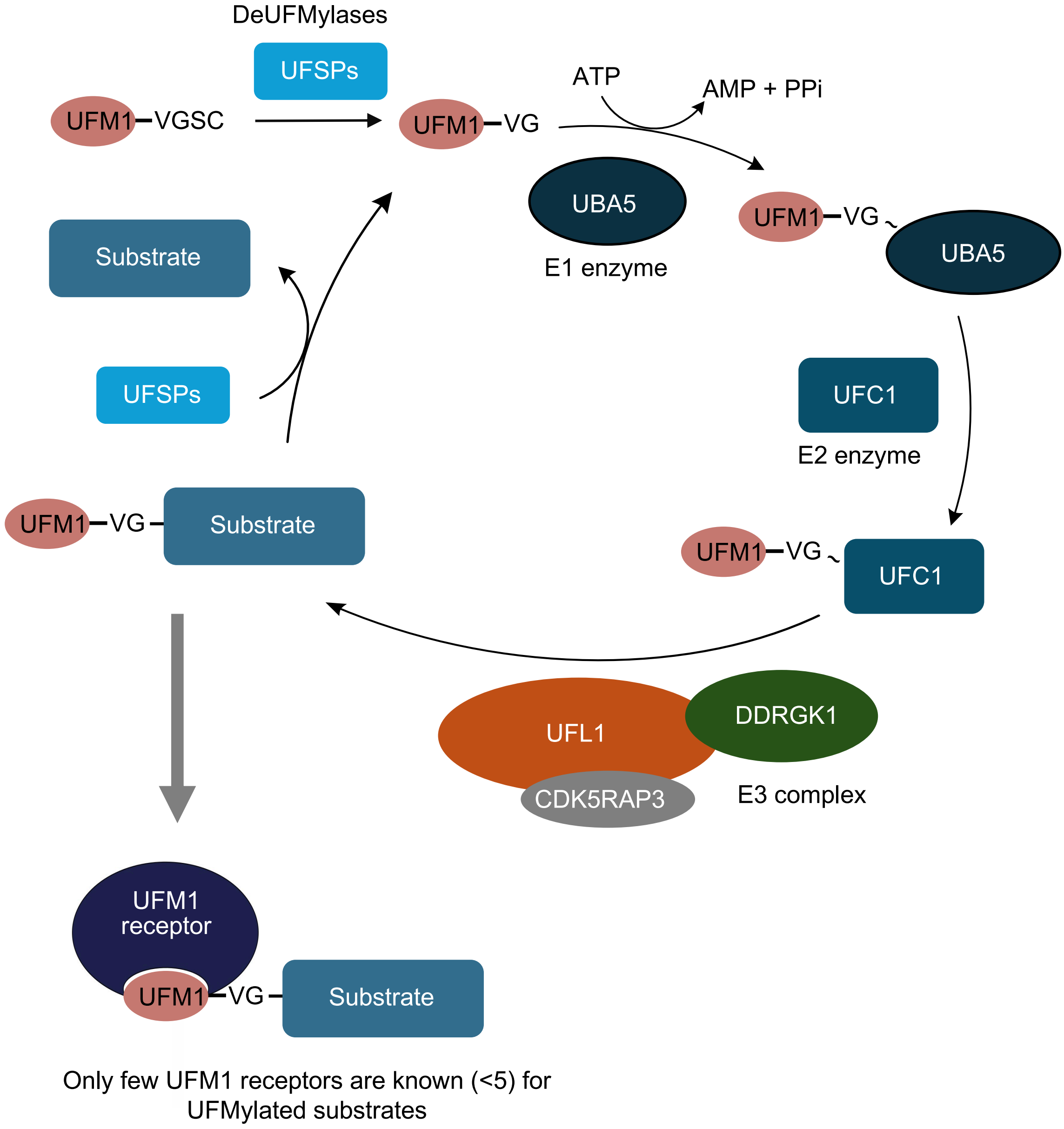

Supplementary Figure 2. Schematic of UFMylation cycle. UFM1 is expressed as an 85-amino acid precursor that is processed via the action of UFSP deUFMylating enzymes to expose the C-terminal glycine that is adenylated by the action of the E1-activating enzyme, UBA5, in an ATP-dependent manner to form a UFM1~UBA5 thioester. Note UFM1 has a Val-Gly conjugatable C-terminus in contrast to ubiquitin, SUMO, ISG15 and other UBLs that have Gly-Gly. UFM1 is transferred to the E2 conjugating enzyme, UFC1, in a transthiolation reaction before transfer of UFM1 to substrate lysine residues through an isopeptide linkage via the E3 complex composed of UFL1, DDRGK1, and CDK5RAP3. UFSP enzymes can remove UFM1 from target substrates to recycle the mature protein. UFMylated substrates need to be read by receptors to translate the modification into a cellular signal or response, with relatively few UFM1 reader proteins or domains known.

Abbreviations: ATP: adenosine triphosphate

Supplementary Figure 3

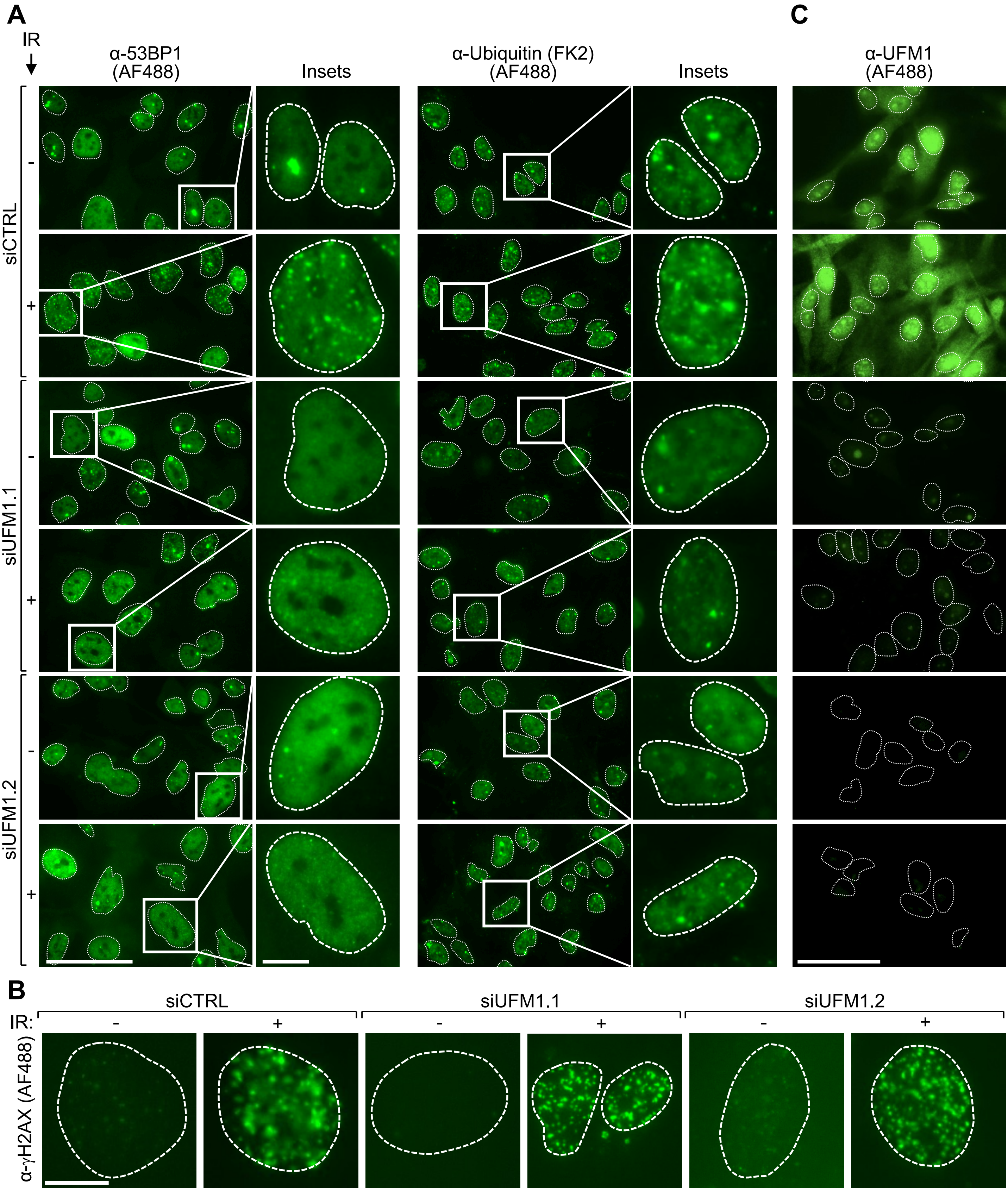

Supplementary Figure 3. UFM1 promotes recruitment of 53BP1 and conjugated ubiquitin to DNA damage sites. Formation of ionising radiation-induced foci (IRIF) of 53BP1 (left) and ubiquitin (FK2 antibody, right) (**A**), as well as γH2AX (**B**), in siUFM1-depleted U2OS cells, using two independent siRNAs (siUFM1.1, siUFM1.2), compared to non-targeting control siRNA (siCTRL, against luciferase). (**C**) siRNA depletion efficiencies of two independent siRNAs targeting UFM1 (siUFM1.1, siUFM1.2), used in (A), as illustrated by diminished immunofluorescent staining in U2OS cells, using an α-UFM1 antibody. Dashed white lines mark nuclei outlines according to DAPI staining. Scale bar represents 50 μm for scaled-out images and 10 μm for insets in (A), 10 μm in (B), and 50 μm in (C).

Abbreviations: α: anti; AF: Alexa Fluor; IRIF: ionising radiation-induced foci.

Supplementary Figure 4

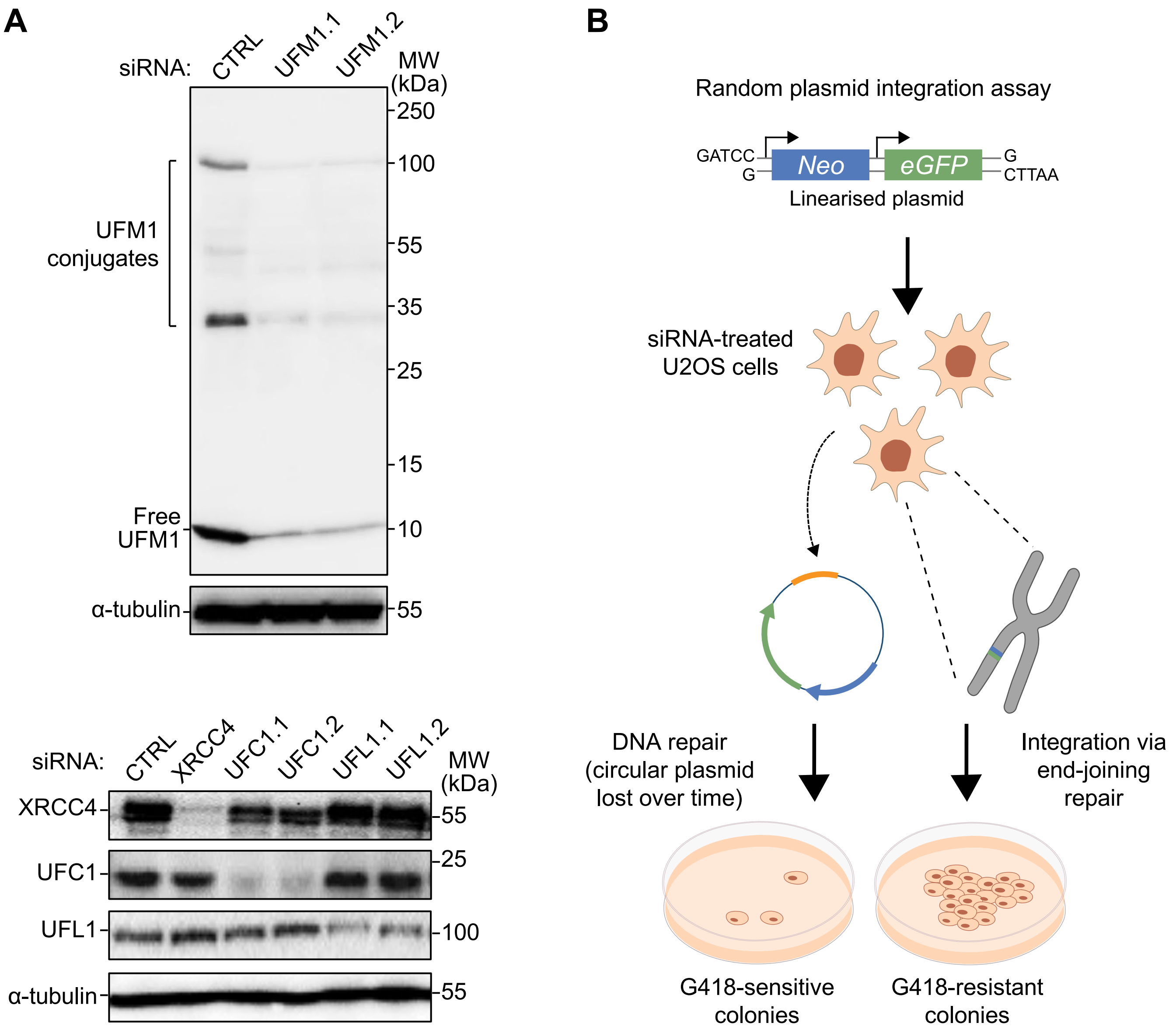

Supplementary Figure 4. Random plasmid integration information for evaluating UFM1 pathway components in end-joining repair. (**A**) siRNA depletion efficiencies assessed by immunoblotting of two independent siRNAs targeting UFM1 (UFM1.1, UFM1.2), UFC1 (UFC1.1, UFC1.2) and UFL1 (UFL1.1, UFL1.2), as well as a positive control, siXRCC4. (**B**) Schematic overview of random plasmid integration assay. Cells were transfected with a linearised plasmid harbouring a neomycin resistance cassette (*Neo*) conferring resistance to geneticin (G418), along with a *GFP* reporter to assess transfection efficiency. Successful G418-resistant colony formation requires genomic integration of the *Neo* cassette via end-joining repair, enabling quantification of surviving colonies as a functional readout of end-joining repair efficiency.

Abbreviations: α: anti; MW: molecular weight; Neo: neomycin resistance cassette.

Supplementary Figure 5

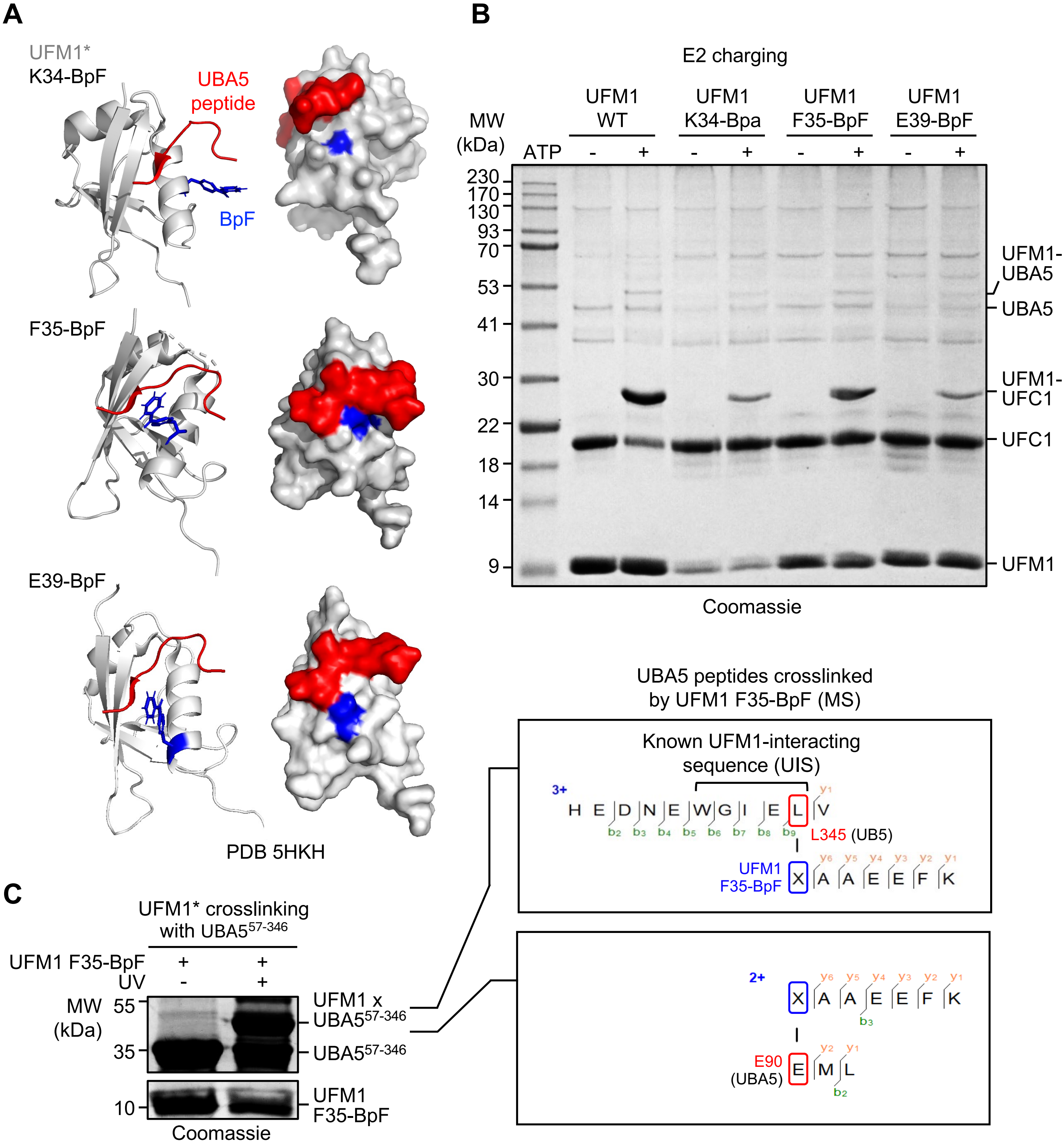

Supplementary Figure 5. Establishment of a photo-crosslinkable UFM1 probe for capturing weak and transient interactors with its α-β groove. (**A**) Ribbon and surface representation of BpF incorporation into UFM1 (PBD 5HKH) at K34, F35, and E39 relative to binding of the UFM1-interacting sequence (UIS, amino acids 338-346) from UBA5. (**B**) E2 charging assays using UFM1, wildtype (WT) or mutants incorporating BpF at K34, F35, or E39, indicating the functional integrity of UFM1 F35-BpF (same gel as in Figure 2C). (**C**) Left: photo-crosslinking reaction (365 nm, 2 h) of UFM1 F35-BpF with recombinant UBA5$^{57\text{-}346}$. Right: two crosslinked peptides obtained from the reaction displayed on the left by mass spectrometry (MS), corresponding to the UIS, and the adenylation domain, of UBA5.

Abbreviations: MS: mass spectrometry; MW: molecular weight, UIS: UFM1-interacting sequence; WT: wildtype.

Supplementary Figure 6

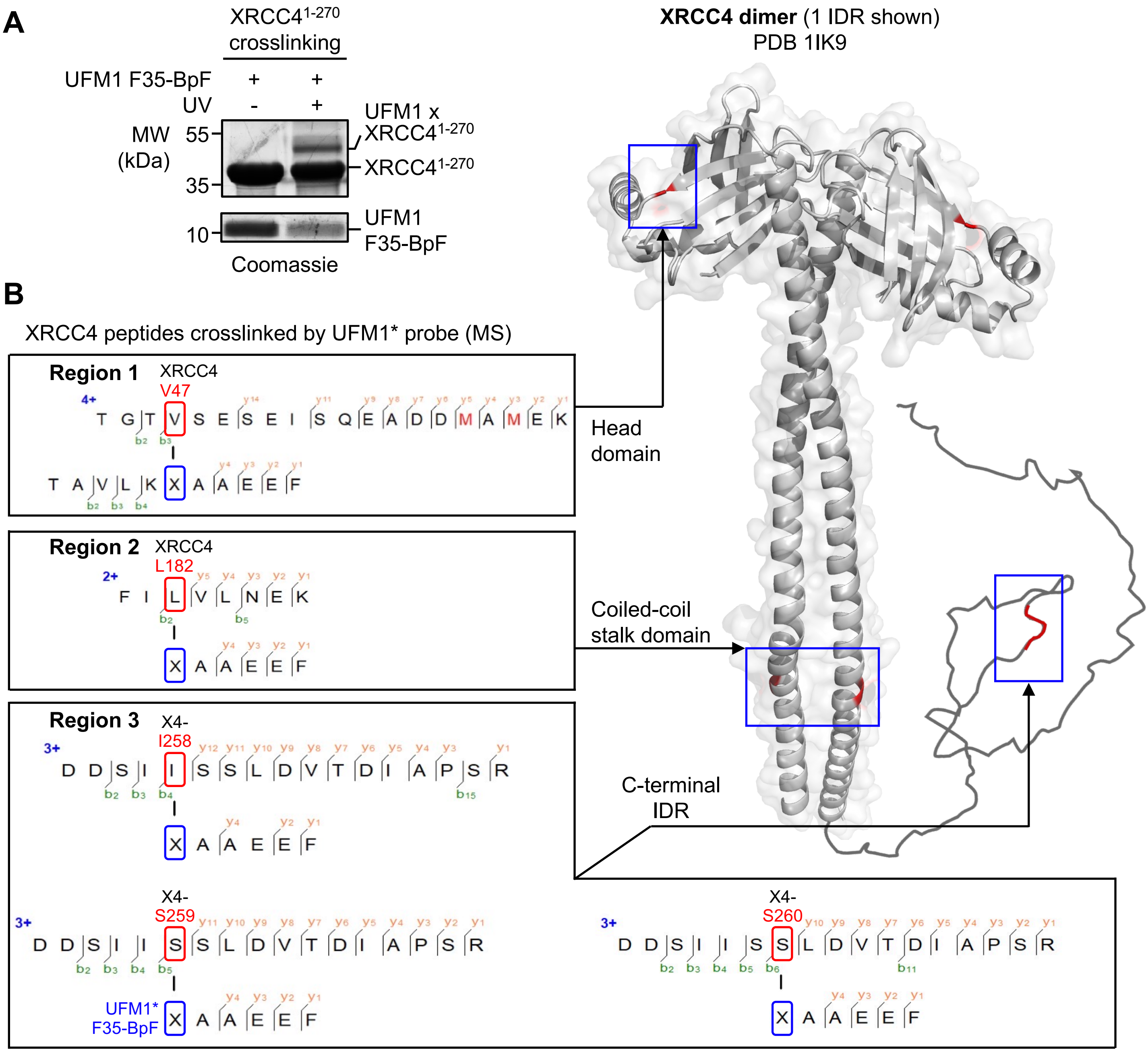

C

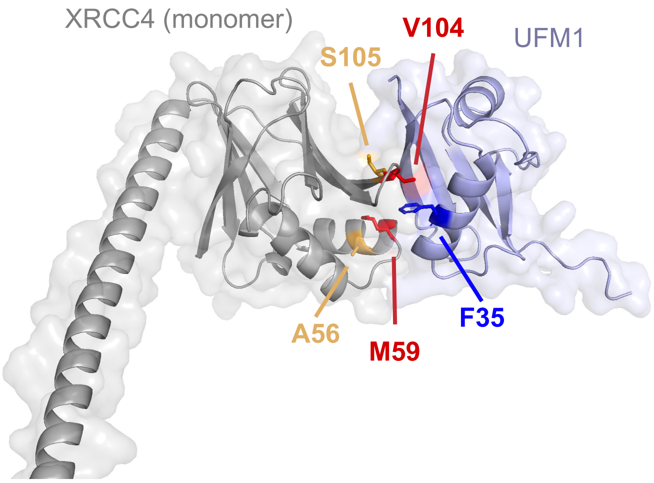

Supplementary Figure 6. Identification of UFM1 crosslinking regions on XRCC4. (**A**) Photo-crosslinking reaction (365 nm, 2 h) of UFM1 F35-BpF with recombinant XRCC4$^{1\text{-}270}$. (**B**) Five crosslinked peptides obtained from the reaction in (A) and identified by mass spectrometry (MS), corresponding to three distinct regions on XRCC4 in the head domain (Region 1), the coiled-coil stalk (Region 2), and the C-terminal intrinsically disordered region (IDR, Region 3), as depicted on the right (PDB 1IK9). Crosslinked residues, valine 47 (V47), leucine 182 (L182), isoleucine 258 (I258) and serines 259 and 260 (S259, S260), are highlighted in red, F35-BpF in blue. (**C**) Modelling the XRCC4-UFM1 interaction with the head domain and coiled coil using AlphaFold3 shows proximity of the F35 residue (where the photo-crosslinkable BpF is incorporated) with V104/M59 (red), and S105/A56 (orange) residues that show significant CSP/intensity loss in NMR experiments (Figure 4A). AlphaFold3 model is deposited in ModelArchive under accession code ma-3oq3s: (https://modelarchive.org/doi/10.5452/ma-3oq3s)

Abbreviations: IDR: intrinsically disordered region; MS: mass spectrometry; MW: molecular weight; UFM1*: UFM1 F35-BpF; X4: XRCC4.

A

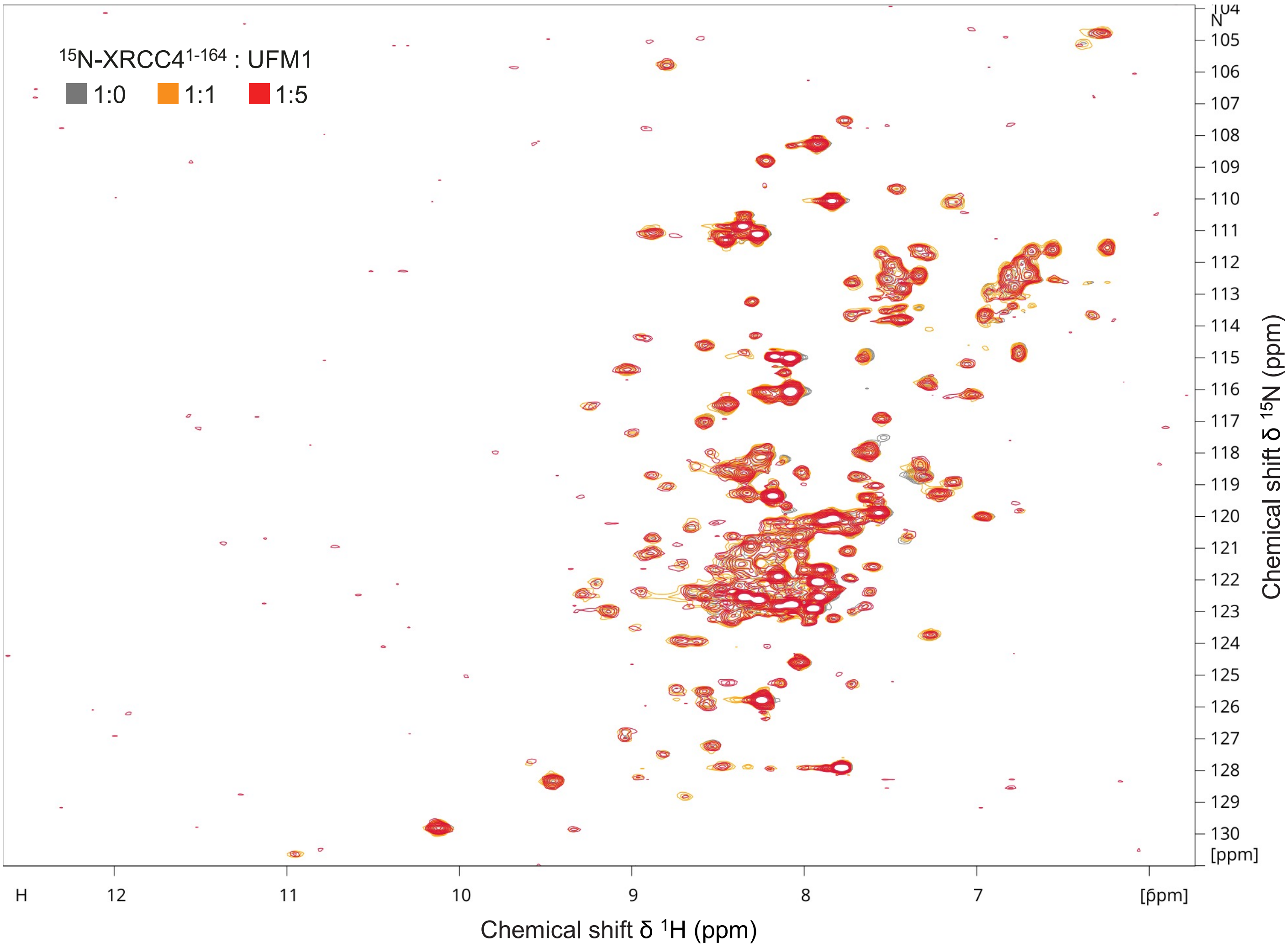

B

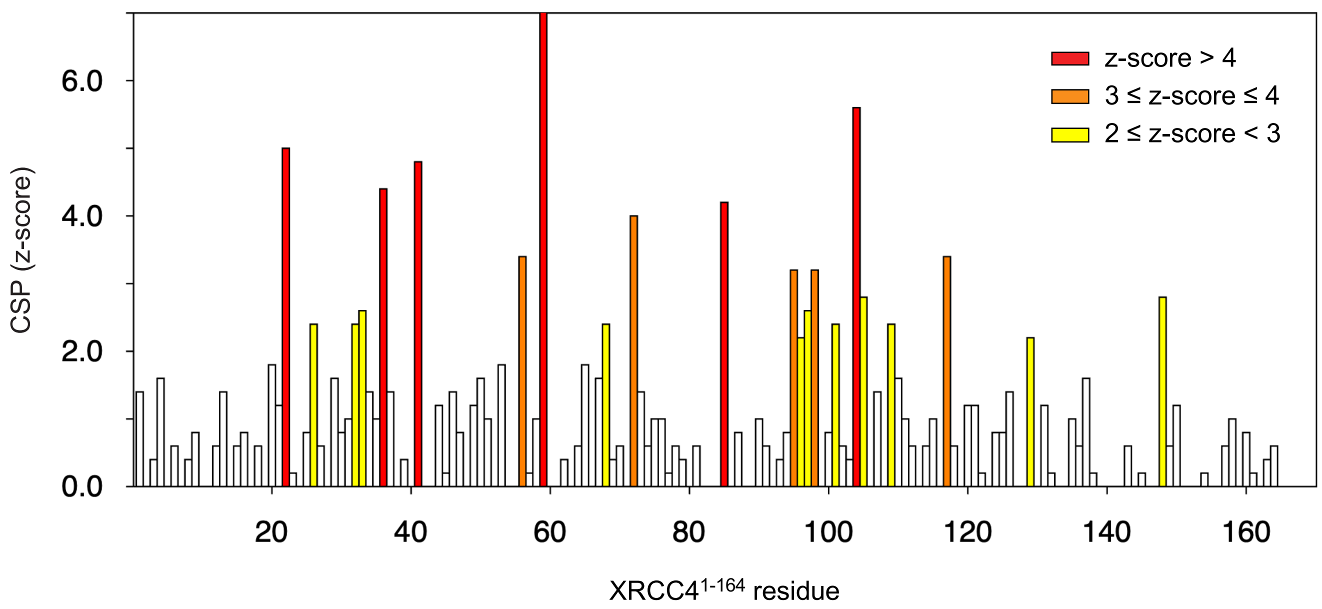

Supplementary Figure 7. NMR spectra and perturbations of XRCC4$^{1\text{-}164}$ binding to UFM1. (**A**) Overlay of ^{1}H-^{15}N BEST-TROSY spectra of XRCC4$^{1\text{-}164}$ alone (grey, 100 µM) and after adding 1 (orange, 100 µM) or 5 (red, 500 µM) equivalents of UFM1. (**B**) Z-scores of chemical shift perturbations (CSPs) extracted from the spectra shown in (A).

Supplementary Figure 8

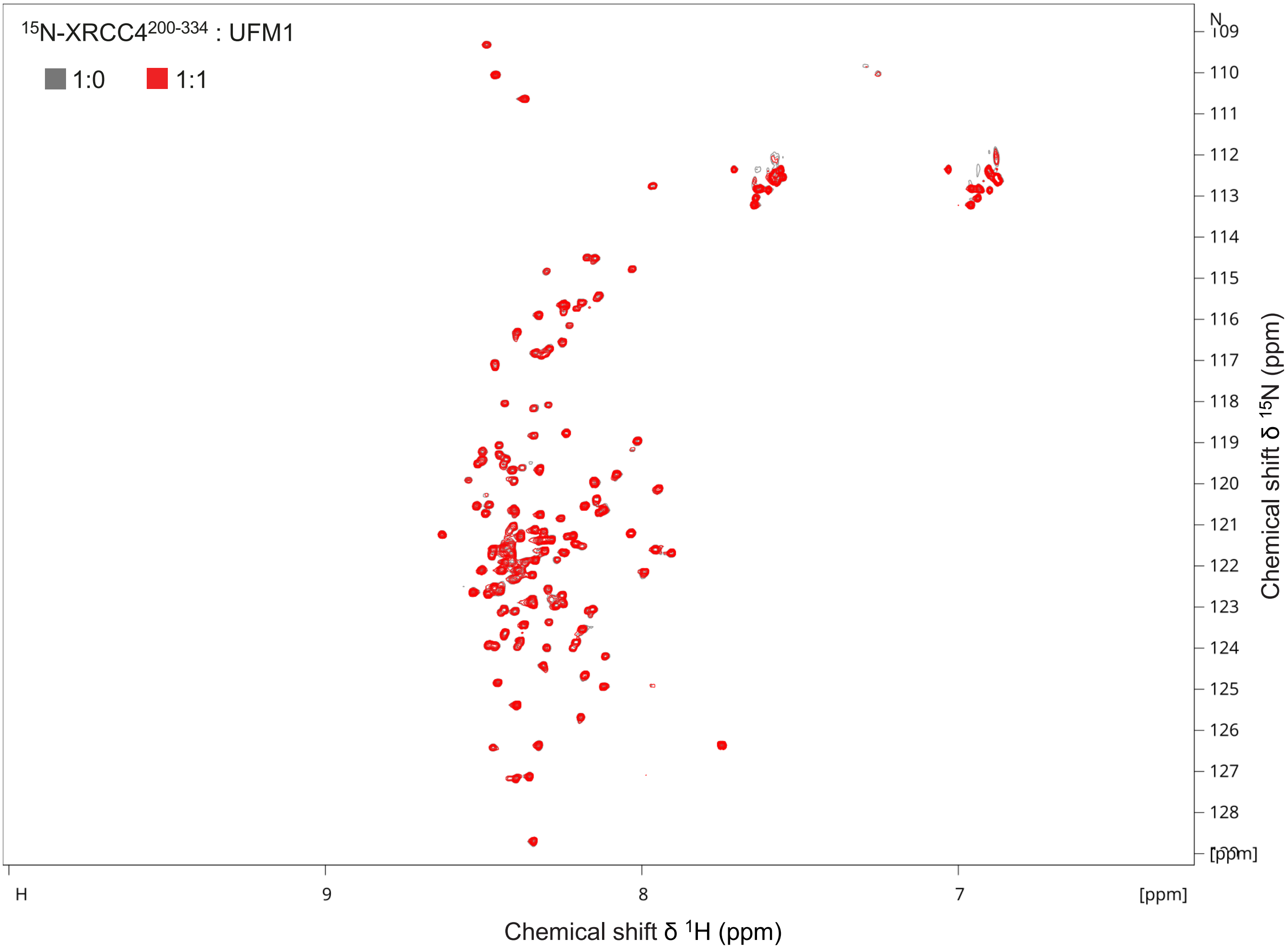

Supplementary Figure 8. NMR spectra of XRCC4$^{200-334}$ binding to UFM1. Overlay of ^{1}H-^{15}N HSQC spectra of XRCC4$^{200-334}$ alone (grey, 195 μM) and in the presence of 1 equivalent of UFM1 (red, 195 μM).

Supplementary Figure 9

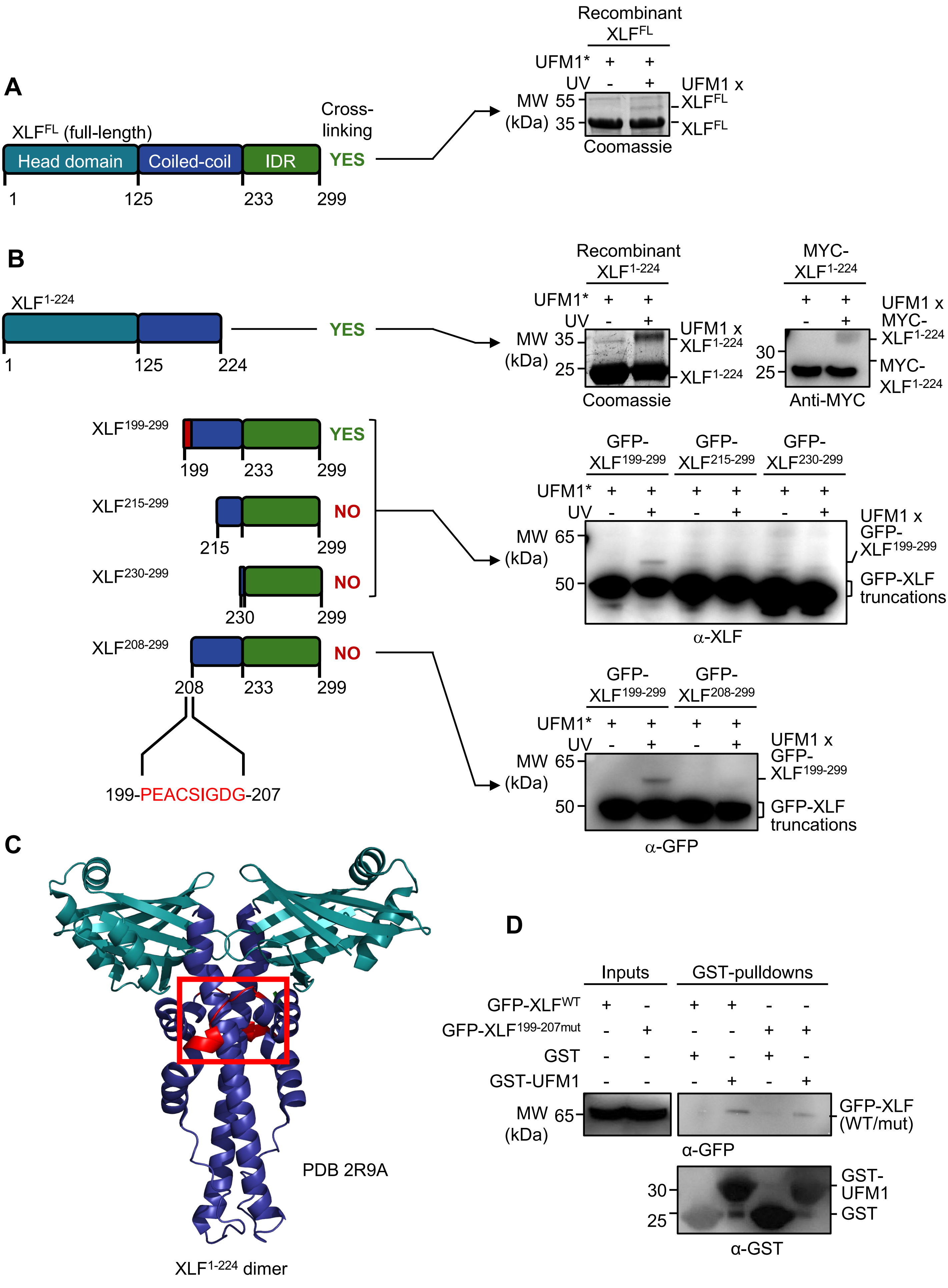

Supplementary Figure 9. **Photo-crosslinking validation of XLF as a UFM1-interacting protein**. (**A**) Full-length purified XLF photo-crosslinks to the UFM1 F35-BpF probe (UFM1*). (**B**) Photo-crosslinking of UFM1* with XLF$^{1\text{-}224}$, either purified from *E. coli* or in HEK293T cell lysate following ectopic expression (top right two panels). Photo-crosslinking of UFM1* with HEK293T cell lysates transiently transfected with GFP-tagged XLF truncation mutants highlights a sequence (199-PEACSIGDG-207) that is required for binding to UFM1* (middle and bottom right panels). Schematics of XLF and the mutants used are shown on the left. (**C**) Structural representation of XLF$^{1\text{-}224}$ dimer (PDB 2R9A), highlighting the location of the UFM1-interacting region (red). (**D**) GST-UFM1 pulldowns with HEK293T cell lysates ectopically expressing GFP-tagged full-length XLF – wildtype (XLF^{WT}) or 199-207 mutated to GGGCGGGS (XLF$^{199\text{-}207mut}$) – highlighting reduced binding to UFM1 when this UFM1-interacting sequence is altered.

Abbreviations: α: anti; MW: molecular weight; UFM1*: UFM1 F35-BpF.

Supplementary Figure 10

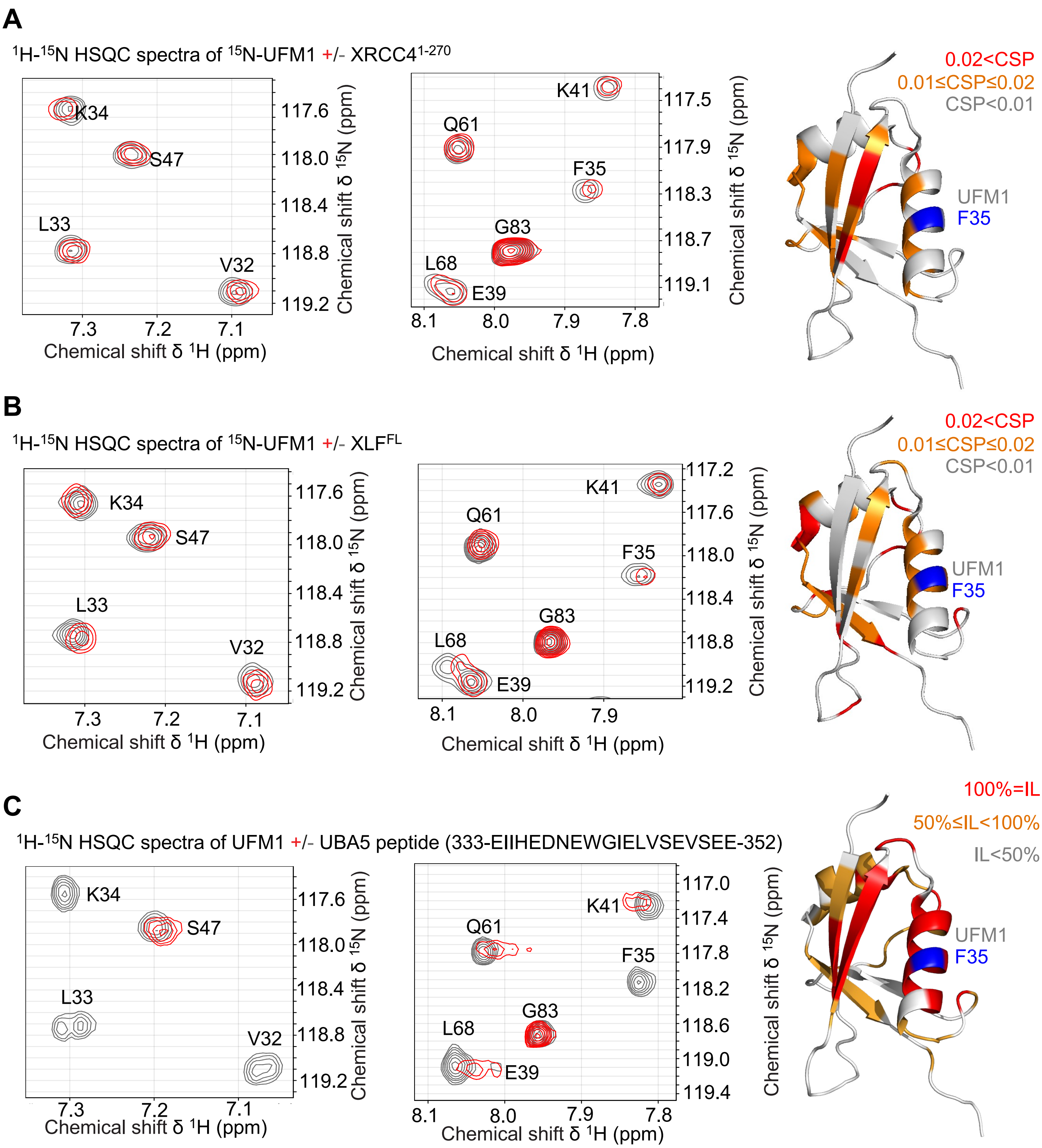

Supplementary Figure 10. XLF and XRCC4 target the α-β groove of UFM1. Left: ^{1}H-^{15}N HSQC spectra of ^{15}N-UFM1 (grey, 100 μM for A and B, 50 μM for C), overlayed with the spectra after the addition of 1 equivalent (red) of XRCC4$^{1\text{-}270}$ (**A**), XLF^{FL} (**B**), or a UBA5 peptide containing the UFM1 interacting sequence (UIS), as indicated in (**C**), showing key residues affected. Right: Corresponding ribbon structures of UFM1 (PBD 5HKH), highlighting the most affected residues by chemical shift perturbation (CSP, XRCC4$^{1\text{-}270}$ and XLF^{FL}) or intensity loss (IL, UBA5 peptide). Colour code ranges from red (residues most affected) over orange (least affected) to grey (not affected), as indicated. F35-BpF is highlighted in blue.

Abbreviations: CSP: chemical shift perturbation; FL: full length; IL: intensity loss; UIS: UFM1-interacting sequence.

Supplementary Figure 11

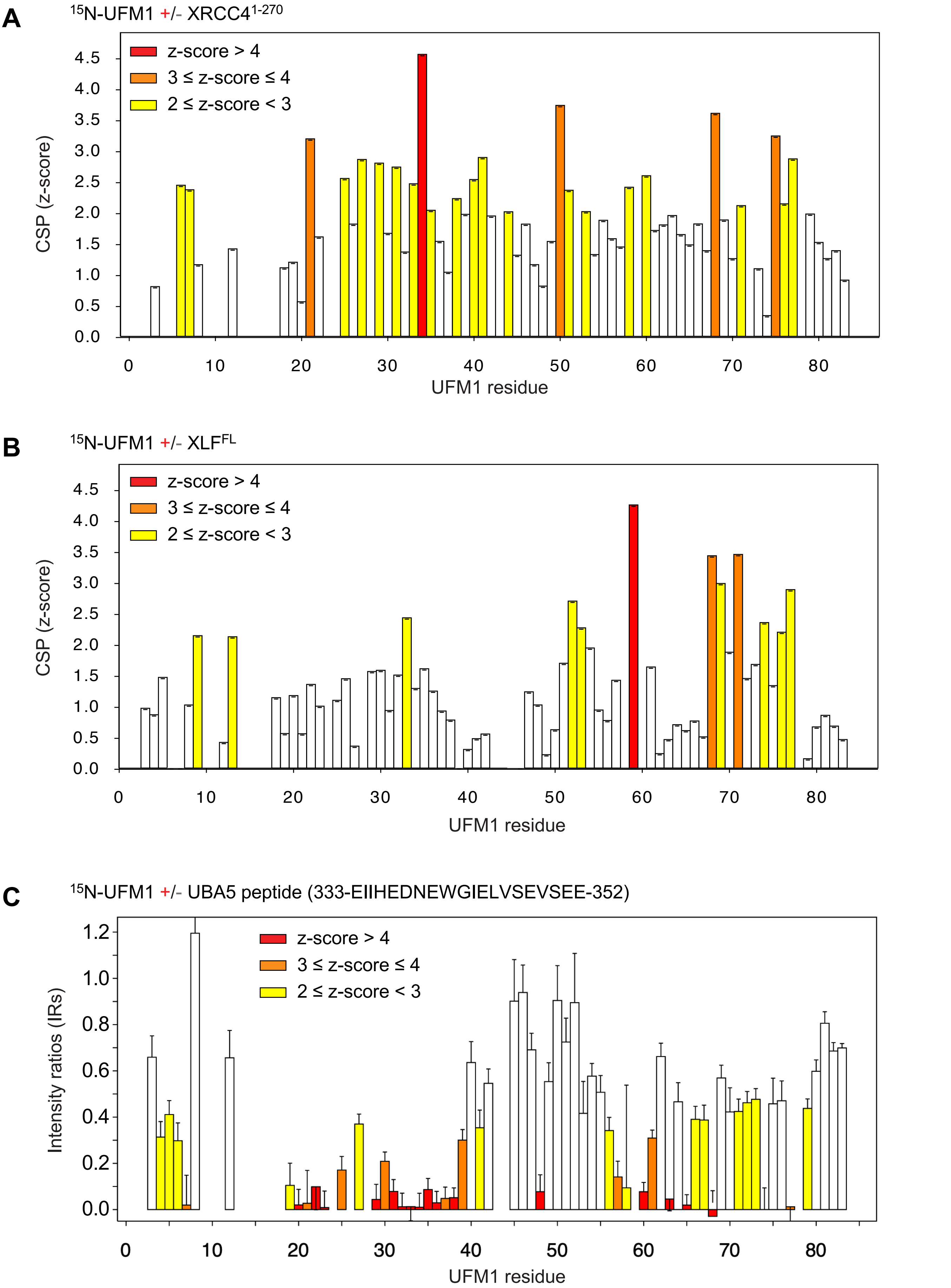

Supplementary Figure 11. Z-scores of chemical shift perturbations (CSPs) extracted from the NMR spectra of ^{15}N-UFM1 binding to (**A**) XRCC4$^{1-270}$ and (**B**) XLF^{FL}. (**C**) Intensity ratios extracted from the NMR spectra of ^{15}N-UFM1. Z-scores relate to CSPs. Error bars represent 1 standard deviation propagated from the baseline deviation.

Supplementary Figure 12

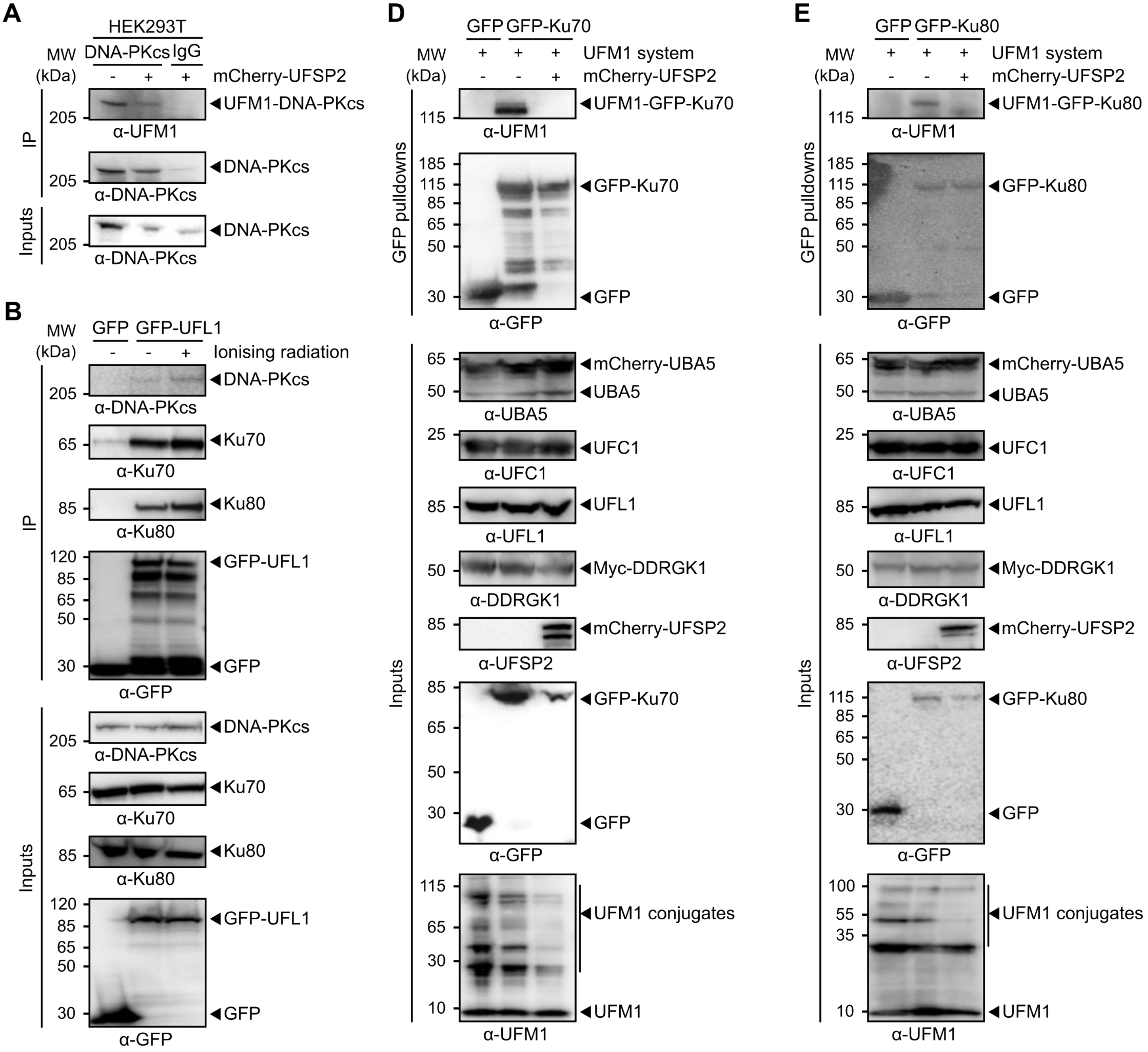

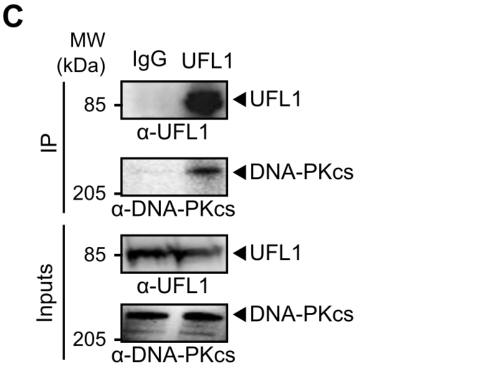

Supplementary Figure 12. UFMylation of DNA-PK complex components. (**A**) Immunoprecipitation (IP) of DNA-PKcs from whole cell extracts, showing a slowly migrating band detectable by a-UFM1 consistent with UFMylation of DNA-PKcs, reduced in the presence of ectopically expressed deUFMylase UFSP2. Whole cell extracts were obtained from HEK293T cells. (**B**) Interaction between UFL1 and the DNA-PK holoenzyme, as shown by GFP-UFL1 pulldown co-precipitating endogenous DNA-PKcs, Ku70 and Ku80. Whole cell extracts were obtained from HEK293T cells, treated, or not, with ionising radiation (IR, ~30 min post 10 Gy). (**C**) Immunoprecipitation (IP) of endogenous UFL1 showing co-precipitation with endogenous DNA-PKcs in whole cell extracts obtained from HEK293T cells. (**D**) GFP-pulldowns of Ku70 from whole cell extracts, showing an a-UFM1-detectable band migrating at a molecular weight consistent with Ku70 UFMylation, which was reduced in the presence of ectopically expressed deUFMylase UFSP2. Whole cell extracts were obtained from HEK293T cells, ectopically expressing GFP-Ku70 and UFM1 system components (UBA5, UFC1, UFL1, DDRGK1) together, or not, with UFSP2. (**E**) As (D) but for GFP-Ku80.

Abbreviations: α: anti; IP: immunoprecipitation; IR: ionising radiation, MW: molecular weight; WT: wildtype.

Supplementary Table 1. List of reagents and resources used.

REAGENT OR RESOURCE	SOURCE	IDENTIFIER
**Antibodies**		
UFM1	Abcam	Cat#ab109305; RRID:AB_10864675
UBA5	Bethyl	Cat#A304-115A; RRID:AB_2621364
UFC1	Abcam	Cat#ab189252
UFL1	Bethyl	Cat#A303-456A; RRID:AB_10951658
DDRGK1/UFBP1	ProteinTech	Cat#21445-1-AP; RRID:AB_2827383
UFSP2	ProteinTech	Cat#16999-1-AP; RRID:AB_2214070
XRCC4	Santa Cruz	Cat#sc-271087; RRID:AB_10612396
Ku70	ProteinTech	Cat#10723-1-AP; RRID:AB_2218756
Ku80	ProteinTech	Cat#16389-1-AP; RRID:AB_2257509
LIG4	Abcam	Cat#ab193353; RRID:AB_2801534
XLF	Cell Signalling Technology	Cat#2854; RRID:AB_2152954
XLF	Bethyl	Cat#A300-730A; RRID:AB_533458
γH2AX	Merck Millipore	Cat#05-636; RRID:AB_309864
α-tubulin	eBioscience	Cat#14-4502-80; RRID:AB_1210457
β-actin	Sigma Aldrich	Cat#A3854; RRID:AB_262011
β-actin	Sigma Aldrich (for EJ7-GFP assays)	Cat#A2066; RRID:AB_476693
H3-HRP	Cell Signalling Technology	Cat#12648; RRID:AB_2797978
HA	Biolegend	Cat#MMS-101R; RRID:AB_291262
GFP	Roche	Cat#11814460001; RRID:AB_390913
GFP	Cell Signalling Technology	Cat#2955; RRID:AB_1196614
DNA-PKcs	Santa Cruz	Cat#sc-5282; RRID:AB_2172848
FLAG	ProteinTech	Cat#20543-1-AP; RRID:AB_11232216
FLAG	Sigma Aldrich (for EJ7-GFP assays)	Cat#A8592; RRID:AB_439702

His_6	Biolegend	Cat#652501; RRID:AB_11204080
Streptavidin-HRP	Thermo Fisher Scientific	Cat#43-4323
Goat anti-rabbit	Invitrogen	Cat#31462
Goat anti-mouse	Dako	Cat#P0260; RRID:AB_2636929
53BP1	Novus Biologicals	Cat#NB100-034
Ubiquitin (FK2)	Enzo Lifesciences	Cat#BML-PW8810; RRID:AB_10541840
AF488 goat anti-rabbit	Invitrogen	Cat#A11034; RRID:AB_2576217
AF594 goat anti-mouse	Invitrogen	Cat#A11032; RRID:AB_2534091
AF594 streptavidin	Thermo Fisher Scientific	Cat#S32356
**Bacterial strains**		
XL1-blue		N/A
BL21 (DE3)		N/A
**Chemicals, peptides, and recombinant proteins**		
UBA5 peptide 333-EIIHEDNEWGIELVSEVSEE-352	Genosphere Biotech	This study
DNA-PKi NU7441	Tocris Bioscience	Cat# 3712
ATMi KU55933	Cambridge Bioscience	Cat#HY-12016-50mg
DNA-PKi AZD7648	Cambridge Bioscience	Cat#HY-111783-5mg
ATMi AZD1390	Cambridge Bioscience	Cat#HY-109566-1mg
G418 (Geneticin)	Gibco	Cat#10131027
Neutravidin agarose	Thermo Fisher Scientific	Cat#29200
Streptavidin sepharose	Cytiva	Cat#17-5113-01
Trimethoprim (TMP)	MP Biomedicals	Cat#T795615
dTagV1	Tocris	Cat#6914
Calicheamicin	MedChem Express	Cat#HY-19609
Ouabain	Tocris	Cat#1076
GFP-Trap beads	Proteintech	Cat#gtma-20
Lipofectamine RNAiMAX	Life Technologies	Cat#13778075
Lipofectamine 3000	Life Technologies	Cat#L3000008
FuGENE 6	Promega	Cat#E2691
**Critical commercial assays**		
Pierce BCA assay kit	Thermo Fisher Scientific	Cat#23227
Neon Transfection System	Invitrogen	Cat#MPK5000

PCR direct lysis reagent	Viagen Biotech	Cat#302-C
**Deposited data**		
Mass spectrometry proteomics (photo-crosslinking)	This study	PRIDE: PXD069136
Mass spectrometry proteomics (APEX2 proximity labelling)	This study	PRIDE: PXD069185
**Experimental models: cell lines**		
U2OS		RRID:CVCL_0042
U2OS *UFSP2-DHFR-FKBP12*	This study	N/A
U2OS *XRCC4* KO	Michał Malewicz	N/A
U2OS TLR system		N/A
HEK293T		RRID:CVCL_0063
HEK293T *XLF/XRCC4* KO	Katheryn Meek[98,99]	N/A
HEK293 *XRCC4/XLF* KO EJ7-GFP	Jeremy Stark[64]	N/A
CB18-0280 fibroblasts (normal)	This study	N/A
CB24-0108 fibroblasts (*UBA5* p.A371T)	Shane McKee[83]	N/A
**siRNA oligonucleotides**		
See Supplementary Data 3		
**Recombinant DNA**		
See Supplementary Data 3		
**Software and algorithms**		
RStudio	https://www.r-project.org/	v 4.2.3; RRID:SCR_000432
LIMMA	https://bioconductor.org/packages/release/bioc/html/limma.html	v 3.54.2; RRID:SCR_010943
GraphPad Prism	https://www.graphpad.com/	v 10.4.2; RRID:SCR_002798
pLink2.0	https://pfind.ict.ac.cn/	v 2.0 and v 3.0.16
Fiji/ImageJ	https://imagej.net/software/fiji/	v 1.54p; RRID:SCR_002285
MaxQuant	https://www.maxquant.org/	v 1.5.2.8; RRID:SCR_014485
Scaffold PTM	Proteome Software	v 4.0.2; RRID:SCR_014345

Spectronaut	Biognosys	17
CometScore 2.0	http://rexhoover.com/index.php?id=cometscore	v 2.0
Pymol	https://pymol.org/	v 3.1; RRID: SCR_000305
ChimeraX	https://www.cgl.ucsf.edu/chimerax/	v 1.9; RRID:SCR_015872
AlphaFold	https://alphafoldserver.com/	
TopSpin (Bruker)	https://www.bruker.com/en/products-and-solutions/mr/nmr-software/topspin.html	v 3.5; RRID:SCR_014227
CCPN AnalysisAssign (v3.2.12)	https://ccpn.ac.uk/software/analysisassign/	v 3.2.12; RRID:SCR_016984
