# Supplementary material for: Systematic discovery of UFM1 receptors reveals a regulatory module in DNA repair directing non-homologous end-joining
Source: Nat Commun. 2026 Jun 15;17:7574. doi: 10.1038/s41467-026-73882-8 (PMC13415529; doi:10.1038/s41467-026-73882-8)
Supplement: Supplementary file 2 — Description of Additional Supplementary Files [file 41467_2026_73882_MOESM2_ESM.pdf]

Description of Additional Supplementary Files

Supplementary Data 1
Mass spectrometry data for UFM1 F35-BpF probe receptor screen.

Supplementary Data 2
Mass spectrometry data for APEX2-UFC1 proximity labelling screen.

Supplementary Data 3
Lists of plasmids and siRNAs used in this study.
