# Supplementary material for: Systematic discovery of UFM1 receptors reveals a regulatory module in DNA repair directing non-homologous end-joining
Source: Nat Commun. 2026 Jun 15;17:7574. doi: 10.1038/s41467-026-73882-8 (PMC13415529; doi:10.1038/s41467-026-73882-8)
Supplement: Supplementary file 6 — Reporting Summary [file 41467_2026_73882_MOESM6_ESM.pdf]

nature portfolio

Corresponding author(s): Dr Christine Schmidt, Dr Matthew Cliff

Last updated by author(s): Mar 12, 2026

Reporting Summary

Nature Portfolio wishes to improve the reproducibility of the work that we publish. This form provides structure for consistency and transparency in reporting. For further information on Nature Portfolio policies, see our Editorial Policies and the Editorial Policy Checklist.

Statistics

For all statistical analyses, confirm that the following items are present in the figure legend, table legend, main text, or Methods section.

n/a	Confirmed	
☐	☒	The exact sample size (*n*) for each experimental group/condition, given as a discrete number and unit of measurement
☐	☒	A statement on whether measurements were taken from distinct samples or whether the same sample was measured repeatedly
☐	☒	The statistical test(s) used AND whether they are one- or two-sided<br>*Only common tests should be described solely by name; describe more complex techniques in the Methods section.*
☒	☐	A description of all covariates tested
☐	☒	A description of any assumptions or corrections, such as tests of normality and adjustment for multiple comparisons
☐	☒	A full description of the statistical parameters including central tendency (e.g. means) or other basic estimates (e.g. regression coefficient) AND variation (e.g. standard deviation) or associated estimates of uncertainty (e.g. confidence intervals)
☒	☐	For null hypothesis testing, the test statistic (e.g. *F*, *t*, *r*) with confidence intervals, effect sizes, degrees of freedom and *P* value noted<br>*Give P values as exact values whenever suitable.*
☒	☐	For Bayesian analysis, information on the choice of priors and Markov chain Monte Carlo settings
☒	☐	For hierarchical and complex designs, identification of the appropriate level for tests and full reporting of outcomes
☒	☐	Estimates of effect sizes (e.g. Cohen's *d*, Pearson's *r*), indicating how they were calculated

Our web collection on statistics for biologists contains articles on many of the points above.

Software and code

Policy information about availability of computer code

Data collection

CellSens software (Evident/Olympus) for image capturing on an Olympus (Evident) Xplore Spin system made up of an Olympus IX83 Confocal microscope body equipped with a Yokogawa CSU-W1 spinning disk. TopSpin (Bruker) was used to collect NMR data on a Bruker 800 MHz spectrometer. Western blot data was collected using ImageLab (Biorad) software. Mass spectrometry data collection was performed using commercially available software developed by the instrument manufacturer: XCalibur (Thermo) on a Vanquish Neo HPLC system coupled to an Orbitrap Lumos mass spectrometer (crosslinking experiments) and on a quadrupole Orbitrap mass spectrometer (Q Exactive Plus, Thermo Scientific) for APEX2 proximity labelling. Acapella (Perkin Elmer) was used to collect high content IRIF data for quantification on a spinning-disk Perkin Elmer Opera platform. Olympus BX51 microscope was used to collect comet assay images.

Data analysis

RStudio https://www.r-project.org/ v 4.2.3; RRID:SCR_000432
LIMMA https://bioconductor.org/packages/release/bioc/html/limma.html v 3.54.2; RRID:SCR_010943
GraphPad Prism https://www.graphpad.com/ v 10.4.2; RRID:SCR_002798
pLink2.0 https://pfind.ict.ac.cn/ v 2.0 and v 3.0.16
Fiji/ImageJ https://imagej.net/software/fiji/ v 1.54p; RRID:SCR_002285
MaxQuant https://www.maxquant.org/ v 1.5.2.8; RRID:SCR_014485
Scaffold PTM Proteome Software v 4.0.2; RRID:SCR_014345
Spectronaut Biognosys 17
CometScore 2.0 http://rexhoover.com/index.php?id=cometscore
Pymol https://pymol.org/ v 3.1; RRID: SCR_000305
Chimerax https://www.cgl.ucsf.edu/chimerax/ v 1.9; RRID:SCR_015872
Alphafold3 https://alphafoldserver.com/

TopSpin (Bruker) https://www.bruker.com/en/products-and-solutions/mr/nmr-software/topspin.html v 3.5; RRID:SCR_014227
CCPN AnalysisAssign (v3.2.12) https://ccpn.ac.uk/software/analysisassign/ v 3.2.12; RRID:SCR_016984

For manuscripts utilizing custom algorithms or software that are central to the research but not yet described in published literature, software must be made available to editors and reviewers. We strongly encourage code deposition in a community repository (e.g. GitHub). See the Nature Portfolio guidelines for submitting code & software for further information.

Data

Policy information about availability of data

All manuscripts must include a data availability statement. This statement should provide the following information, where applicable:

- Accession codes, unique identifiers, or web links for publicly available datasets
- A description of any restrictions on data availability
- For clinical datasets or third party data, please ensure that the statement adheres to our policy

Mass spectrometry proteomics data have been deposited and will be published in full on the PRIDE database (ProteomeXchange) upon acceptance and publication. The UFM1 interaction screen, XL-MS, and Ku UFMylation raw data are currently accessible via the PRIDE database with the identifier PXD069136 and UFC1-APEX2 proximity labelling raw data with the identifier PXD069185. All other data are available in the main text or Supplemental Information. This paper does not report original code. Additional information required to reanalyse the data reported in this paper is available from the lead contact upon reasonable request. Source data have been provided with the manuscript.

Research involving human participants, their data, or biological material

Policy information about studies with human participants or human data. See also policy information about sex, gender (identity/presentation), and sexual orientation and race, ethnicity and racism.

Reporting on sex and gender	N/A; No human research participants were involved in this study.
Reporting on race, ethnicity, or other socially relevant groupings	N/A; No human research participants were involved in this study.
Population characteristics	N/A; No human research participants were involved in this study.
Recruitment	N/A; No human research participants were involved in this study.
Ethics oversight	N/A; No human research participants were involved in this study.

Note that full information on the approval of the study protocol must also be provided in the manuscript.

Field-specific reporting

Please select the one below that is the best fit for your research. If you are not sure, read the appropriate sections before making your selection.

☒ Life sciences ☐ Behavioural & social sciences ☐ Ecological, evolutionary & environmental sciences

For a reference copy of the document with all sections, see nature.com/documents/nr-reporting-summary-flat.pdf

Life sciences study design

All studies must disclose on these points even when the disclosure is negative.

Sample size	No sample size calculations were performed for the experiments included in this paper. We determined that biological replicates would be appropriate for our experiments (when possible) as this would reflect sufficiently on the reproducibility of the results and allow the application of statistical analysis where appropriate.
Data exclusions	No data were excluded.
Replication	All attempts at replicating the data were successful and therefore included in the paper. Proteomics experiments were conducted using technical replicates to ensure robust data generation, in addition to the independent replicates conducted for experiments as outlined in the manuscript.
Randomization	The nature of our study did not require any randomization to be performed.
Blinding	The nature of our study did not require any considerations regarding blinding.

Reporting for specific materials, systems and methods

We require information from authors about some types of materials, experimental systems and methods used in many studies. Here, indicate whether each material, system or method listed is relevant to your study. If you are not sure if a list item applies to your research, read the appropriate section before selecting a response.

Materials & experimental systems

n/a	Involved in the study
☐	☒ Antibodies
☐	☒ Eukaryotic cell lines
☒	☐ Palaeontology and archaeology
☒	☐ Animals and other organisms
☒	☐ Clinical data
☒	☐ Dual use research of concern
☒	☐ Plants

Methods

n/a	Involved in the study
☒	☐ ChIP-seq
☐	☒ Flow cytometry
☒	☐ MRI-based neuroimaging

Antibodies

Antibodies used

UFM1 Abcam Cat#ab109305; RRID:AB_10864675
UBA5 Bethyl Cat#A304-115A; RRID:AB_2621364
UFC1 Abcam Cat#ab189252
UFL1 Bethyl Cat#A303-456A; RRID:AB_10951658
DDRGK1/UFBP1 ProteinTech Cat#21445-1-AP; RRID:AB_2827383
UFSP2 ProteinTech Cat#16999-1-AP; RRID:AB_2214070
XRCC4 Santa Cruz Cat#sc-271087; RRID:AB_10612396
Ku70 ProteinTech Cat#10723-1-AP; RRID:AB_2218756
Ku80 ProteinTech Cat#16389-1-AP; RRID:AB_2257509
LIG4 Abcam Cat#ab193353; RRID:AB_2801534
XLF Cell Signalling Technology Cat#2854; RRID:AB_2152954
XLF Bethyl Cat#A300-730A; RRID:AB_533458
γH2AX Merck Millipore Cat#05-636; RRID:AB_309864
alpha-tubulin eBioscience Cat#14-4502-80; RRID:AB_1210457
beta-actin Sigma Aldrich Cat#A3854; RRID:AB_262011
beta-actin Sigma Aldrich (for EJ7-GFP assays) Cat#A2066; RRID:AB_476693
H3-HRP Cell Signalling Technology Cat#12648; RRID:AB_2797978
HA Biolegend Cat#MMS-101R; RRID:AB_291262
GFP Roche Cat#11814460001; RRID:AB_390913
GFP Cell Signalling Technology Cat#2955; RRID:AB_1196614
DNA-PKcs Santa Cruz Cat#sc-5282; RRID:AB_2172848
FLAG ProteinTech Cat#20543-1-AP; RRID:AB_11232216
FLAG Sigma Aldrich (for EJ7-GFP assays) Cat#A8592; RRID:AB_439702
His6 Biolegend Cat#652501; RRID:AB_11204080
Streptavidin-HRP Thermo Fisher Scientific Cat#43-4323
Goat anti-rabbit Invitrogen Cat#31462
Goat anti-mouse Dako Cat#P0260; RRID:AB_2636929
53BP1 Novus Biologicals Cat#NB100-034
Ubiquitin (FK2) Enzo Lifesciences Cat#BML-PW8810; RRID:AB_10541840
AF488 goat anti-rabbit Invitrogen Cat#A11034; RRID:AB_2576217
AF594 goat anti-mouse Invitrogen Cat#A11032; RRID:AB_2534091
AF594 Streptavidin Thermo Fisher Scientific Cat#S32356

Validation

Antibodies were validated by the manufacturer for applications such as WB, IF or IHC in multiple cell lines and/or different types of human tissues. The manufacturer's website also lists peer-reviewed publications where antibodies have been referred to.

Eukaryotic cell lines

Policy information about cell lines and Sex and Gender in Research

Cell line source(s)

HEK-293T (CVCL_0063), U2OS (CVCL_0042), U2OS XRCC4 knockout (Michał Malewicz, Polish Center for Technology Development, Wroclaw, Poland), CB18-0280 (patient-derived fibroblasts from a healthy male, Stefan Meyer this study), CB24-0108 (UBA5 A371T mutant patient-derived fibroblasts, Shane McKee, Department of Genetic Medicine, Belfast City Hospital, Belfast, UK), U2OS TLR (Stephen Jackson, CRUK Cambridge Institute, UK), U2OS UFSP2-DHFR-FKBP12 (this study), XRCC4 KO and XRCC4/XLF double KO HEK293T cells (Katheryn Meek (Michigan State University, USA), XRCC4/XLF KO HEK293 with EJ7-GFP stably integrated (Jeremy Stark, Beckman Research Institute of the City of Hope, Duarte, CA, USA).

Authentication

For all cell lines, the morphology of the cells was frequently monitored under the microscope to ensure that the culture has its correct and expected appearance.

Mycoplasma contamination

Utilized cell lines for this study have tested negative for mycoplasma contamination.

Commonly misidentified lines
(See ICLAC register)

Our study did not include any cell lines that are commonly misidentified.

Plants

Seed stocks: n/a

Novel plant genotypes: n/a

Authentication: n/a

Flow Cytometry

Plots

Confirm that:

- [ ] The axis labels state the marker and fluorochrome used (e.g. CD4-FITC).
- [ ] The axis scales are clearly visible. Include numbers along axes only for bottom left plot of group (a 'group' is an analysis of identical markers).
- [ ] All plots are contour plots with outliers or pseudocolor plots.
- [x] A numerical value for number of cells or percentage (with statistics) is provided.

Methodology

Sample preparation: U2OS cells stably carrying the TLR cassette were transfected with the indicated siRNAs or treated with small-molecule inhibitors (DNA-PK inhibitor (NU7441, Tocris Bioscience, 3 µM 72 h; proteasome inhibitor MG132, 0.1 µM, 48 h; ATM inhibitor KU55933, 10 µM, 72 h; DNA-PK inhibitor AZD7648, 20 µM, 72 h; ATM inhibitor AZD1390, Cambridge Bioscience, 20 µM, 72 h). Approximately 8 h later, cells were co-transfected with expression plasmids encoding I-SceI and an infrared fluorescent protein (IFP), alongside a donor construct carrying blue fluorescent protein (BFP). Cells were collected ~72 h post-siRNA transfection. For cell cycle analysis, siRNA-treated U2OS cells were fixed in 70% ethanol, incubated with RNase A (250µg/mL) and propidium iodide (10µg/mL) for 30min at 37°C

For EJ7-GFP experiments, cells were seeded on a 24-well dish at 0.5 × 105 cells per well, and transfected the following day with pX330-7a, px330-7b, XRCC4 plasmid (FLAG-XRCC4WT, FLAG-XRCC4180-5A, FLAG-XRCC4del256-263 or empty vector (EV). For the transfection efficiency control, pCAGGS-NZEGFP and pCAGGS-BSKX were used in place of the px330 plasmids. Three days after transfection, cells were analysed

Instrument: BD LSR Fortessa (BD Biosciences) for TLR experiments, FACSCalibur (BD Biosciences) for cell cycle analysis
ACEA Quanteon for EJ7-GFP DNA damage reporter experiments

Software: CellQuest software for cell cycle analysis, FlowJo (FlowJo, LLC)

Cell population abundance: At least 10,000 IFP^+/BFP^+ double-positive cells were recorded per condition.

Gating strategy: Gating strategies for the TLR and EJ7-GFP experiments have been published previously and are referenced appropriately in the text.

- [ ] Tick this box to confirm that a figure exemplifying the gating strategy is provided in the Supplementary Information.
