# Supplementary material for: Systematic discovery of UFM1 receptors reveals a regulatory module in DNA repair directing non-homologous end-joining
Source: Nat Commun. 2026 Jun 15;17:7574. doi: 10.1038/s41467-026-73882-8 (PMC13415529; doi:10.1038/s41467-026-73882-8)

Figure 2

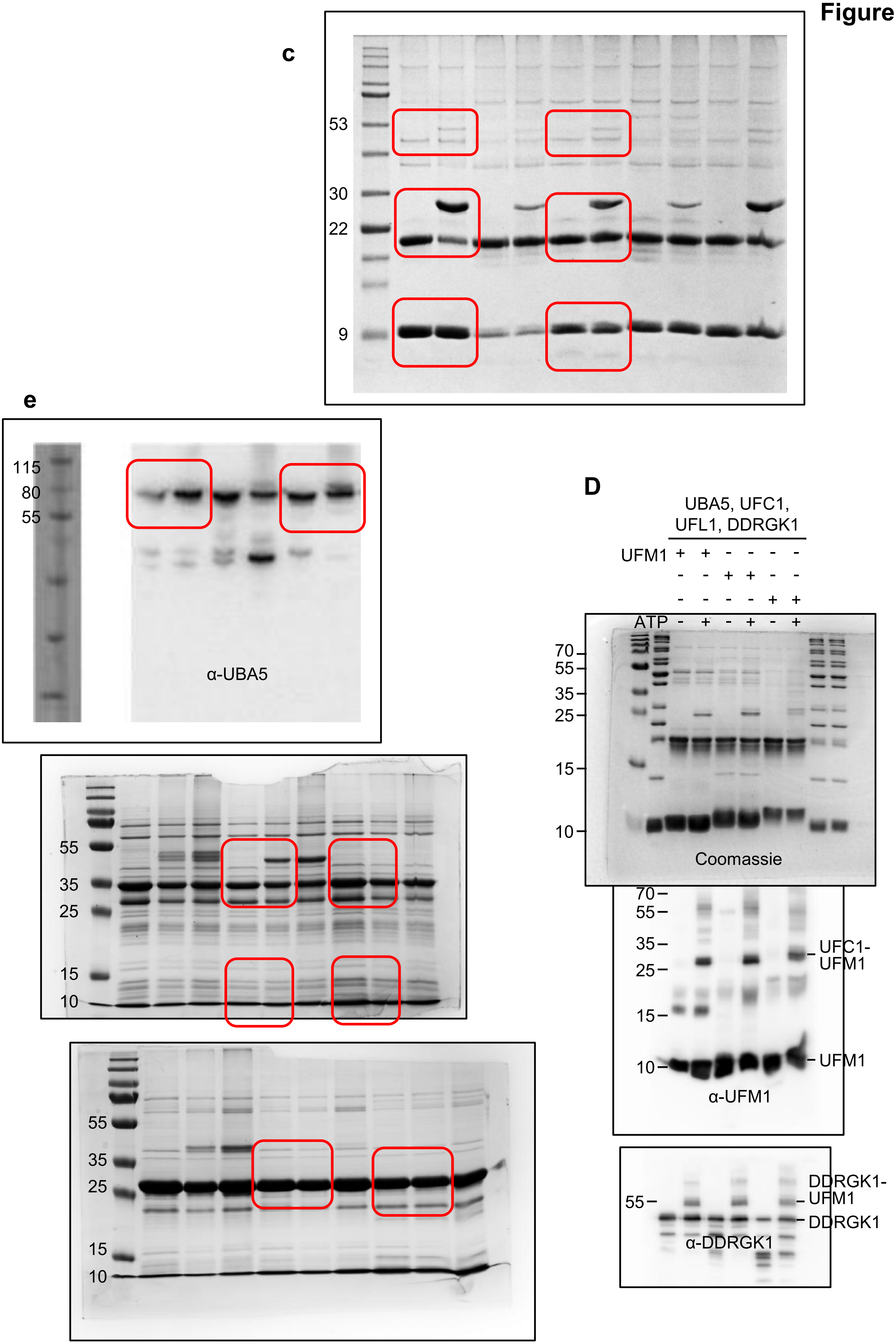

c
53
30
22
9
e
115
80
55
α-UBA5
55
35
25
15
10
55
35
25
15
10
D
UBA5, UFC1,
UFL1, DDRGK1
UFM1 + + - - - -
- - + + - -
- - - - + +
ATP - + - + - +
70
55
35
25
15
10
Coomassie
70
55
35
25
15
10
UFC1-UFM1
UFM1
α-UFM1
55
DDRGK1-UFM1
DDRGK1
α-DDRGK1

Figure 3

c

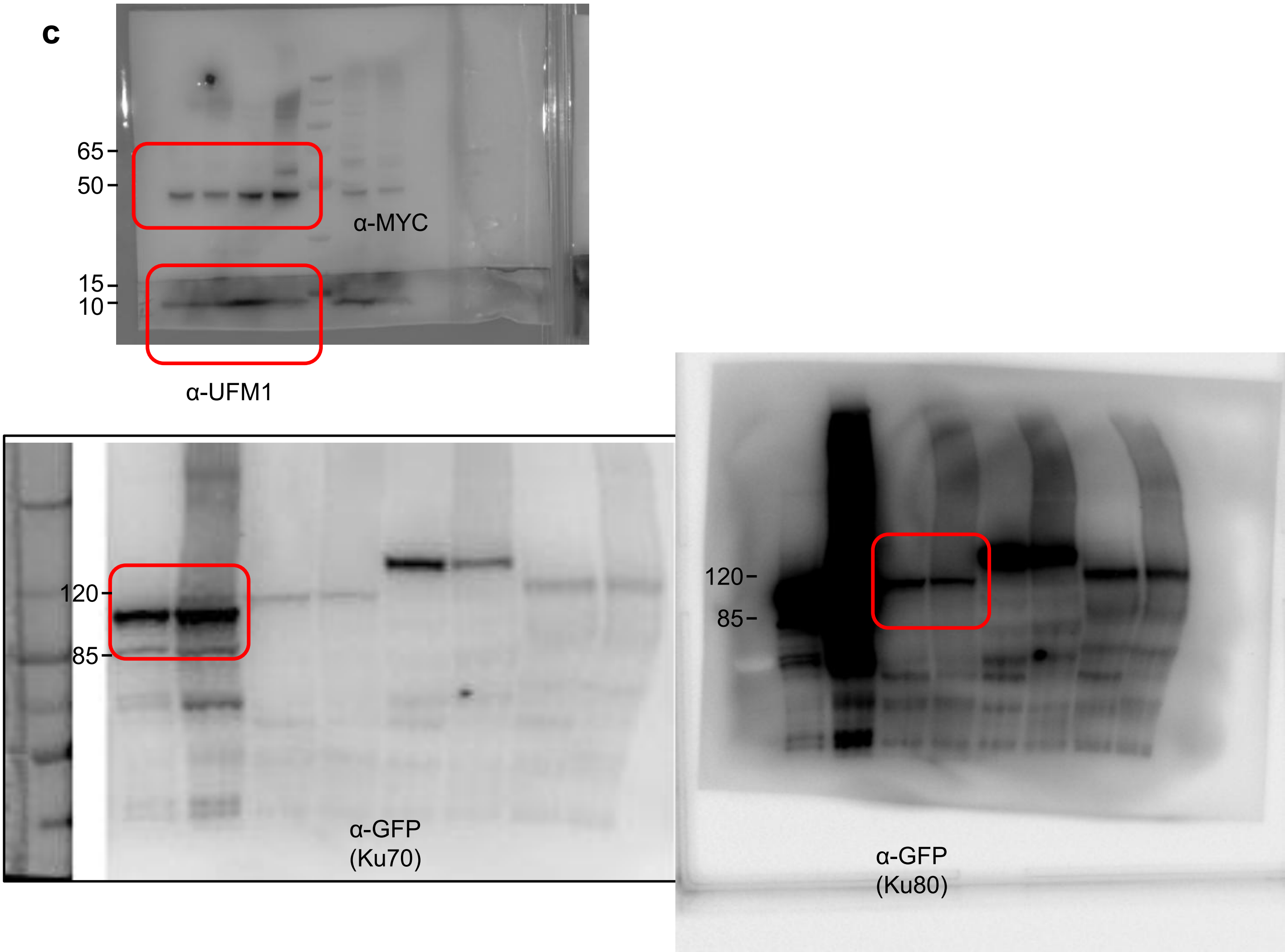
65
50
α-MYC
15
10
α-UFM1
120
85
α-GFP
(Ku70)
120
85
α-GFP
(Ku80)

d

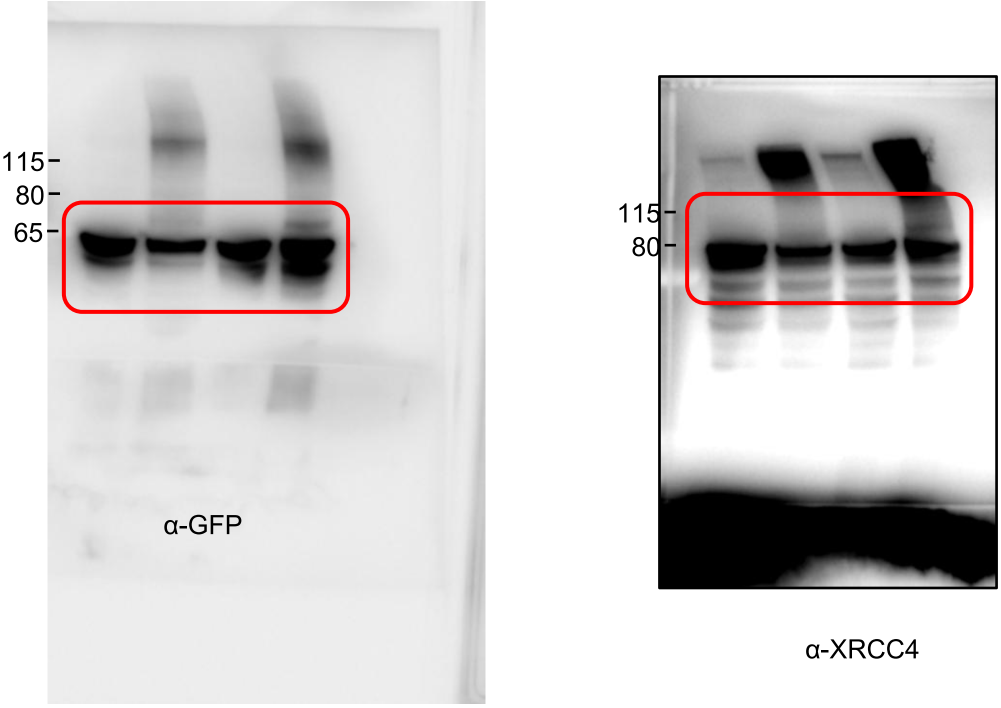
115
80
65
α-GFP
115
80
α-XRCC4

Figure 4

Figure 4C

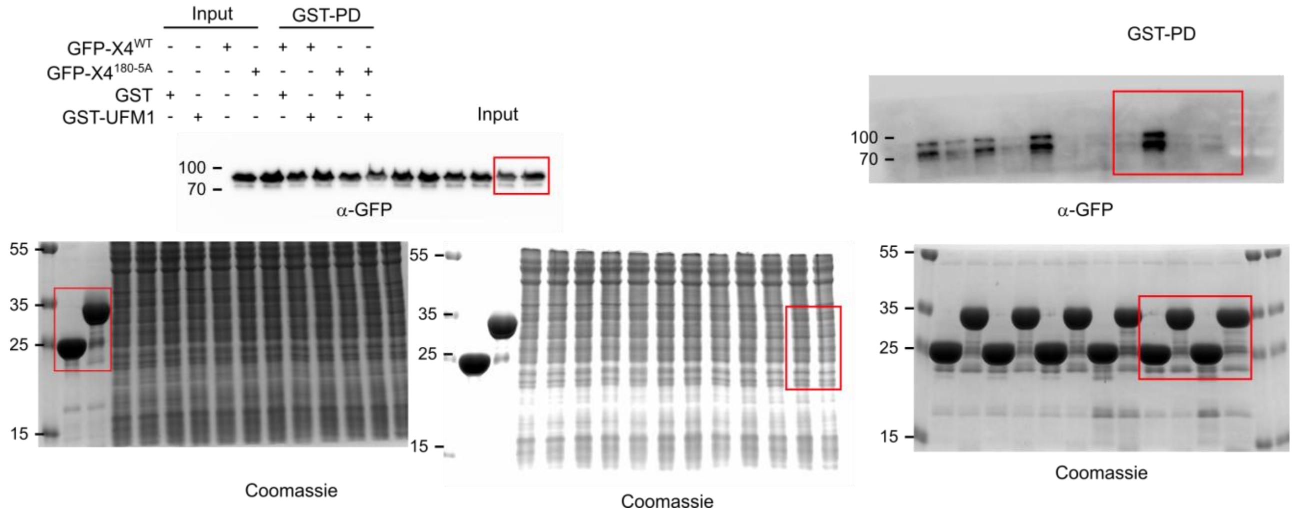

Figure 4E

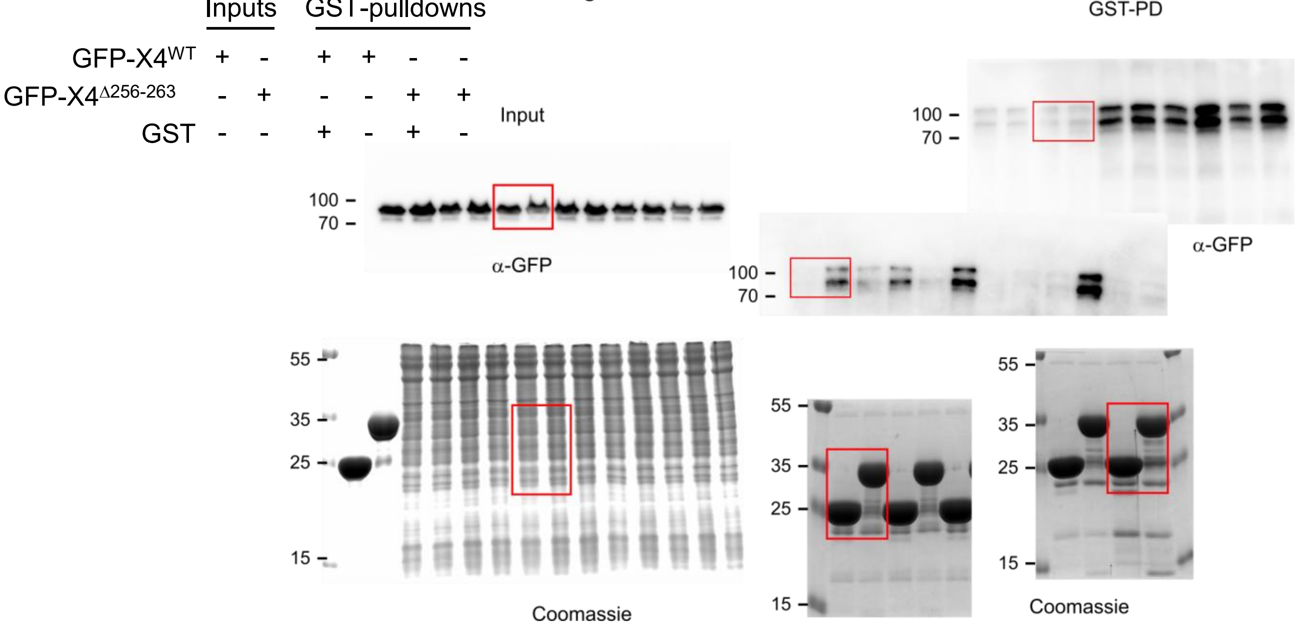

Figure 4F

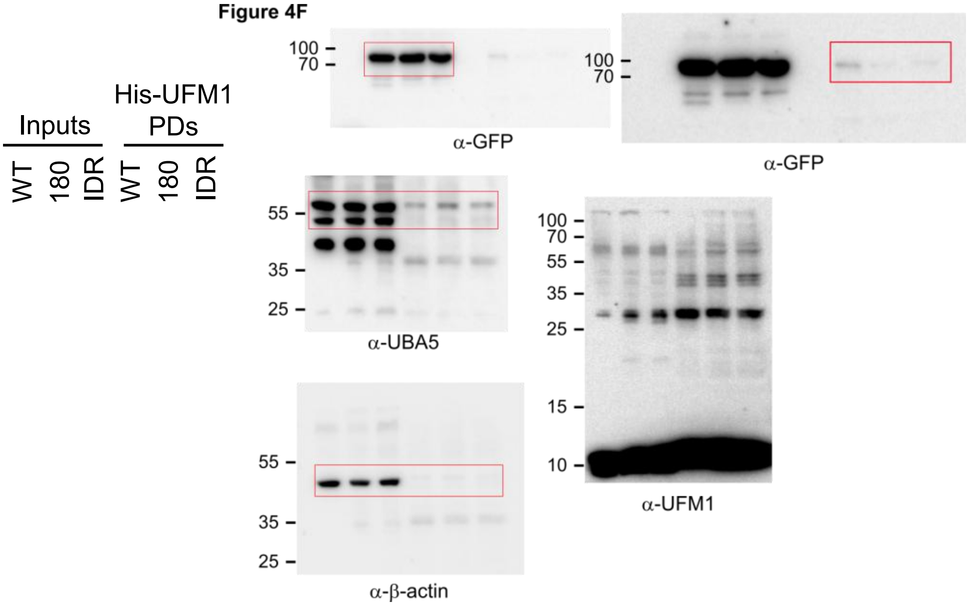

Figure 6

Figure 6A

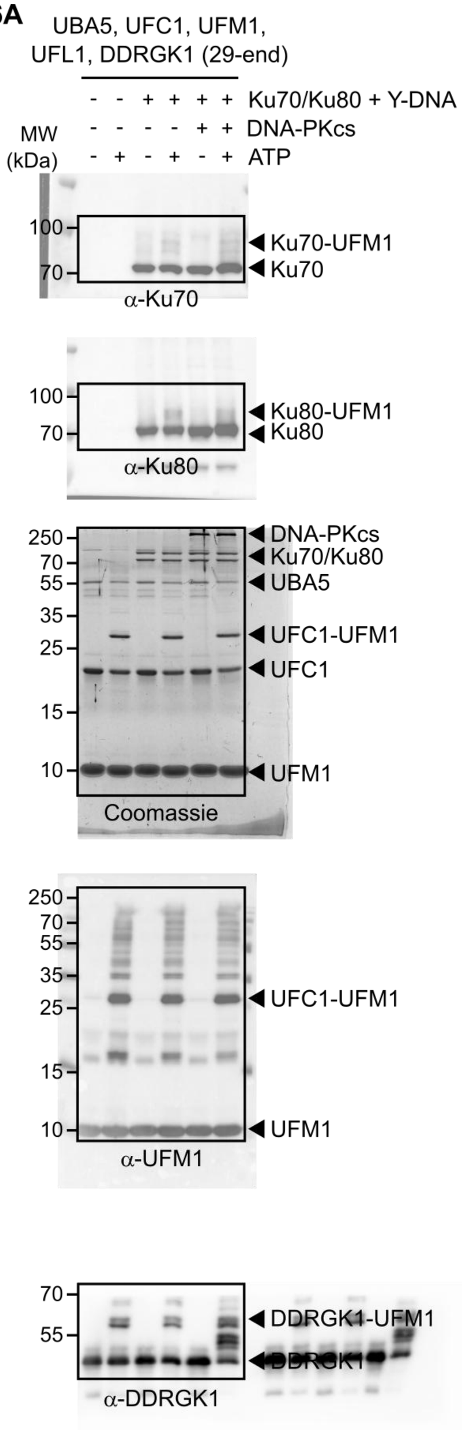

Figure 6F

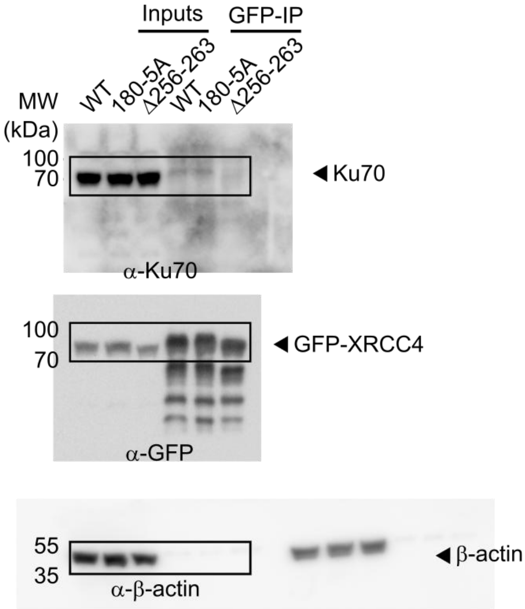

Figure 6

Figure 6C

Input

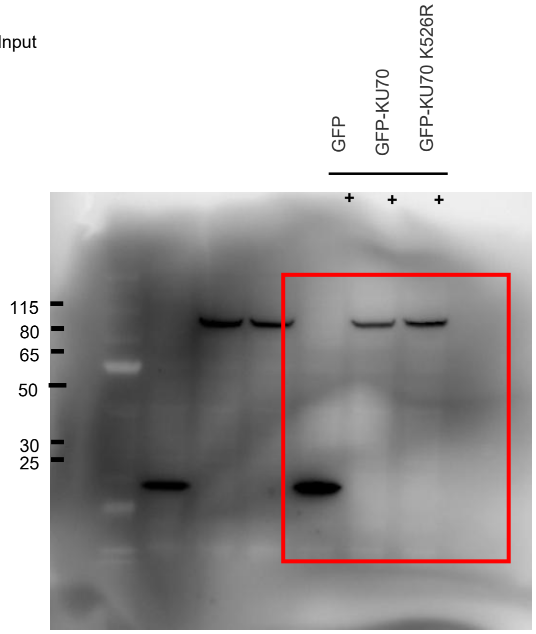

Anti GFP

Pulldown

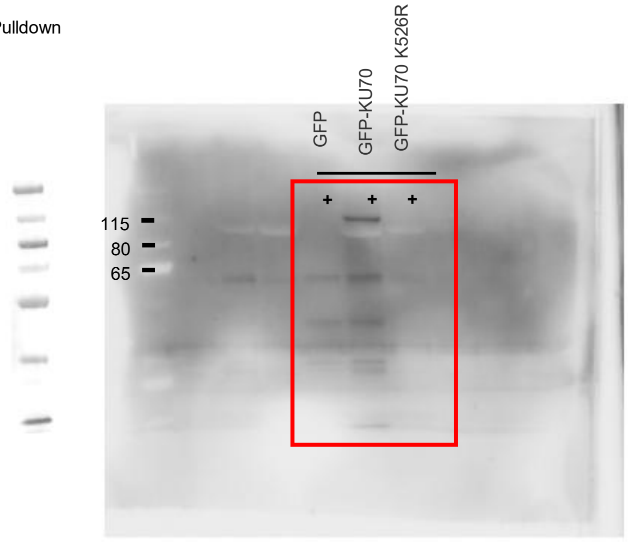

Anti UFM1

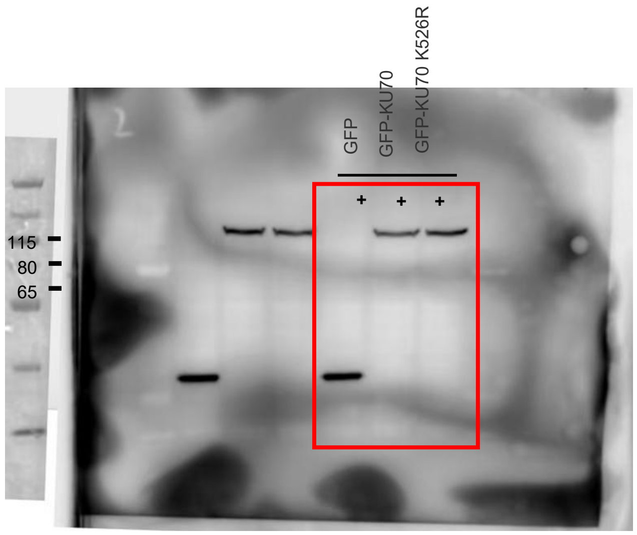

Anti GFP

Figure 6E

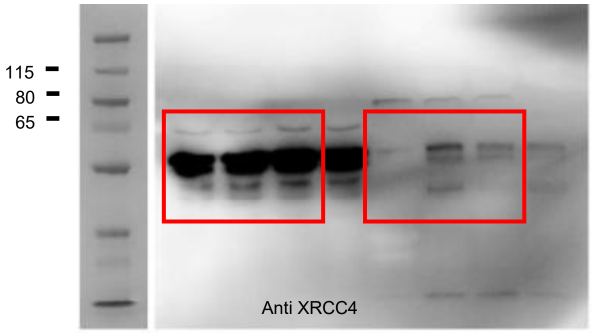

Anti XRCC4

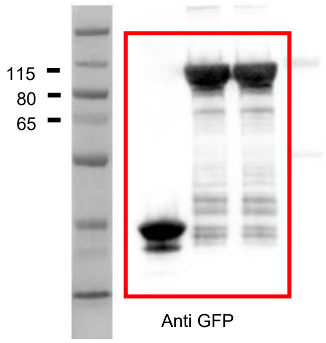

Anti GFP

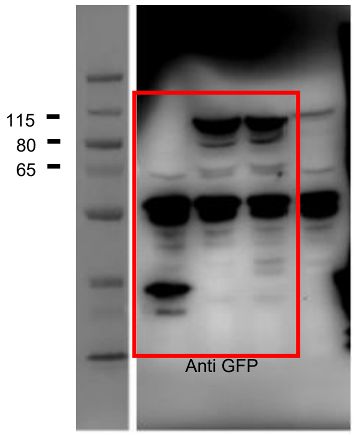

Anti GFP

Figure 7

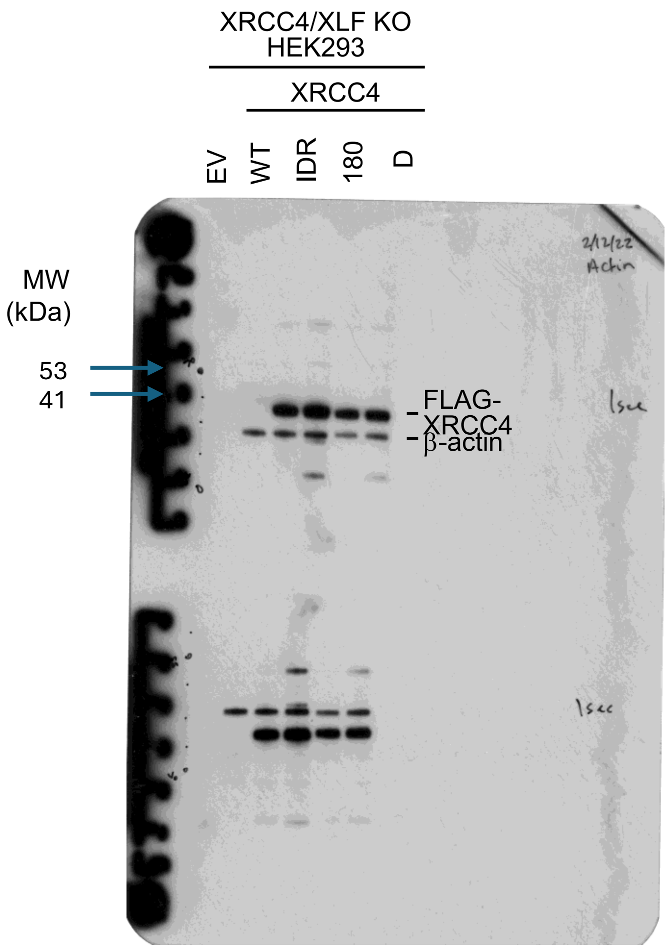
XRCC4/XLF KO
HEK293
XRCC4
EV
WT
IDR
180
D
MW
(kDa)
53
41
FLAG-
XRCC4
β-actin
2/12/22
Actin
1sec
1sec

Figure 8

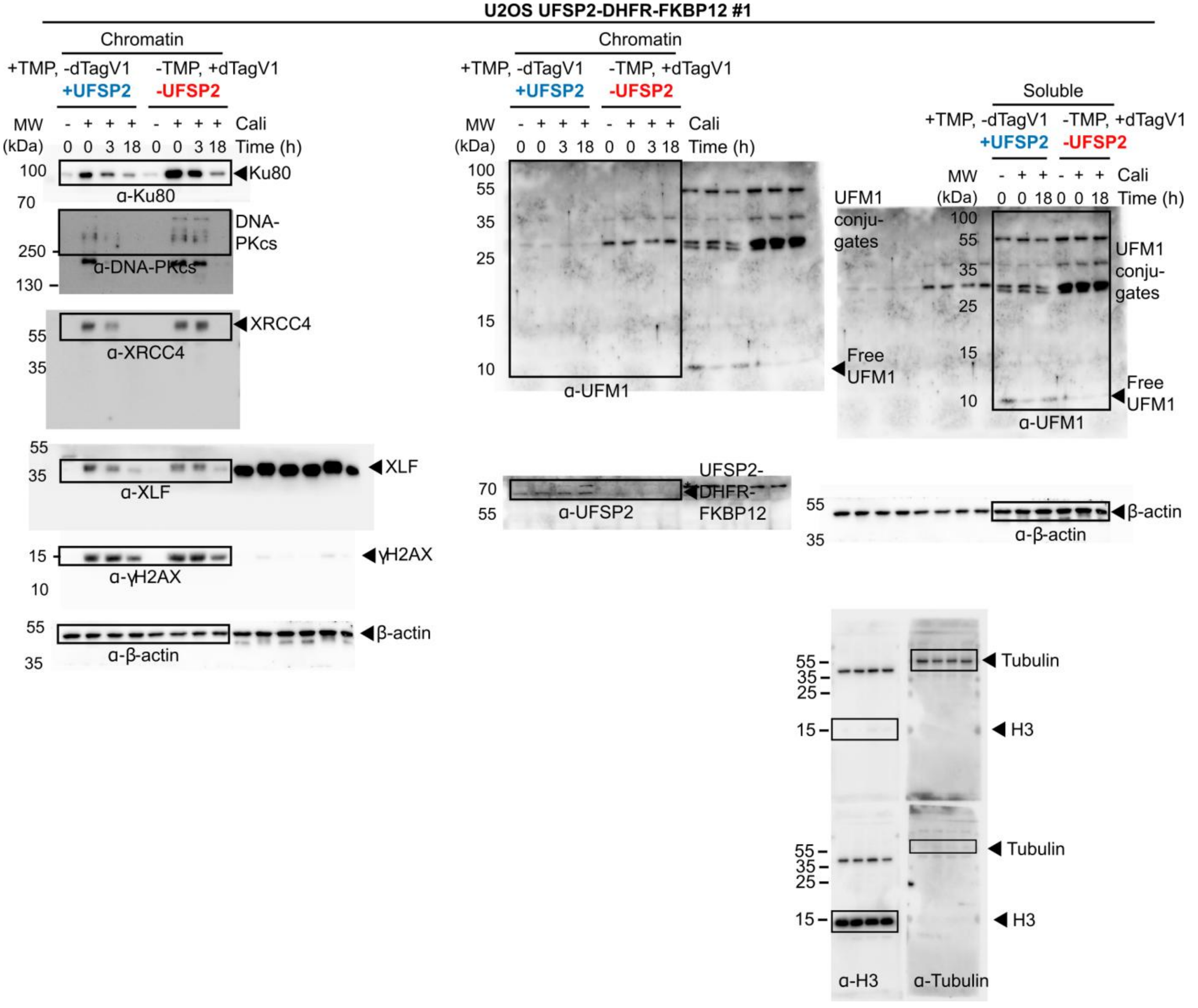
U2OS UFSP2-DHFR-FKBP12 #1
Chromatin
+TMP, -dTagV1
+UFSP2
-TMP, +dTagV1
-UFSP2
MW (kDa)
Cali
Time (h)
Ku80
α-Ku80
DNA-PKcs
α-DNA-PKcs
XRCC4
α-XRCC4
XLF
α-XLF
γH2AX
α-γH2AX
β-actin
α-β-actin
α-UFM1
UFM1 conjugates
Free UFM1
α-UFSP2
UFSP2-DHFR-FKBP12
Soluble
Tubulin
H3
α-H3
α-Tubulin

Figure 9

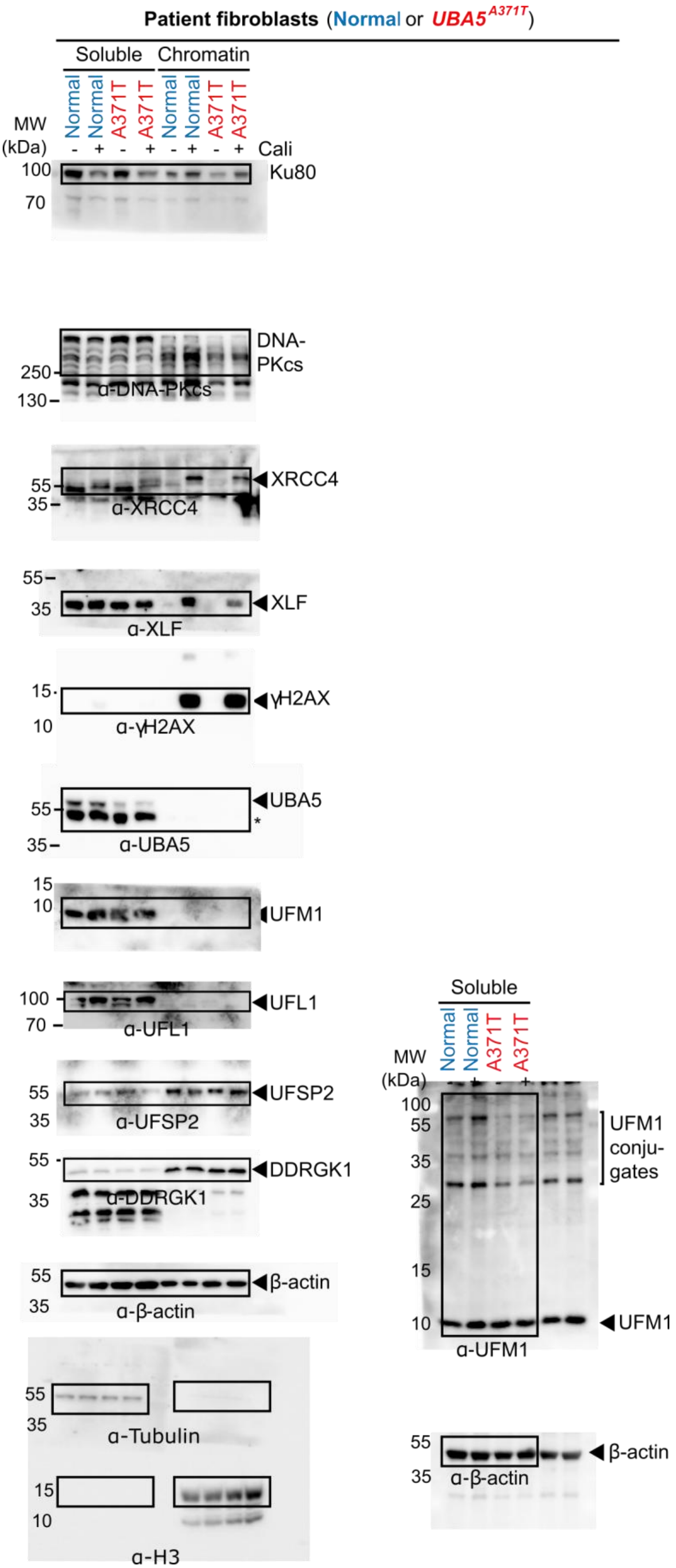
Patient fibroblasts (Normal or UBA5A371T)
Soluble
Chromatin
Normal
Normal
A371T
A371T
Normal
Normal
A371T
A371T
MW (kDa)
- + - + - + - +
Cali
100
70
Ku80
250
130
DNA-PKcs
α-DNA-PKcs
55
35
XRCC4
α-XRCC4
55
35
XLF
α-XLF
15
10
γH2AX
α-γH2AX
55
35
UBA5
*
α-UBA5
15
10
UFM1
100
70
UFL1
α-UFL1
55
35
UFSP2
α-UFSP2
55
35
DDRGK1
α-DDRGK1
55
35
β-actin
α-β-actin
55
35
α-Tubulin
15
10
α-H3
Soluble
Normal
Normal
A371T
A371T
MW (kDa)
- + - +
100
55
35
25
15
10
UFM1 conju-gates
UFM1
α-UFM1
55
35
β-actin
α-β-actin

Supplementary Figure 3

C

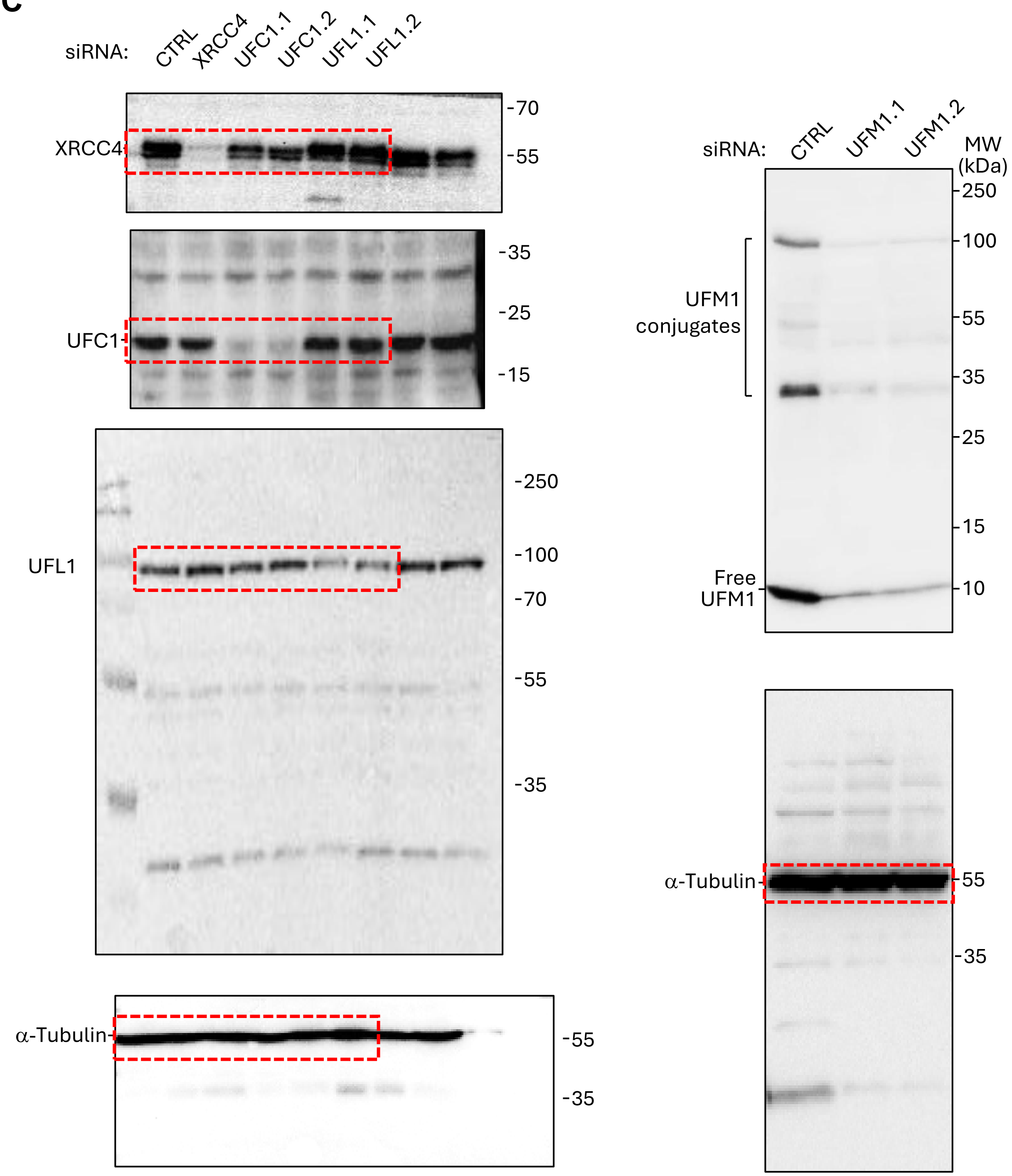

Supplementary Figure 5

B

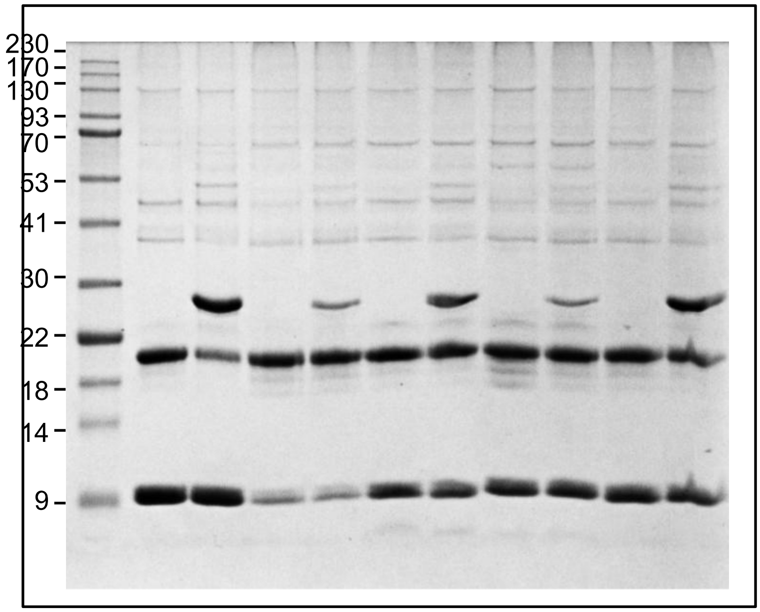

Coomassie

c

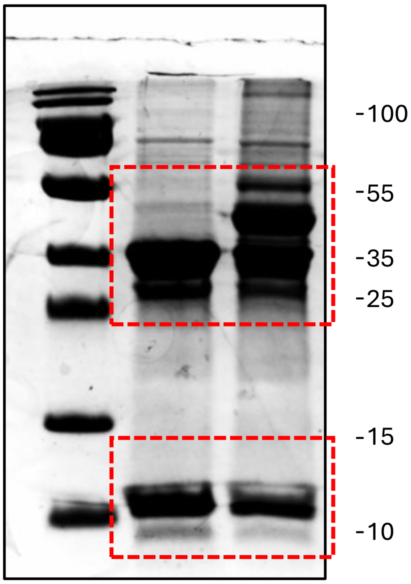

Coomassie

Supplementary Figure 6

a

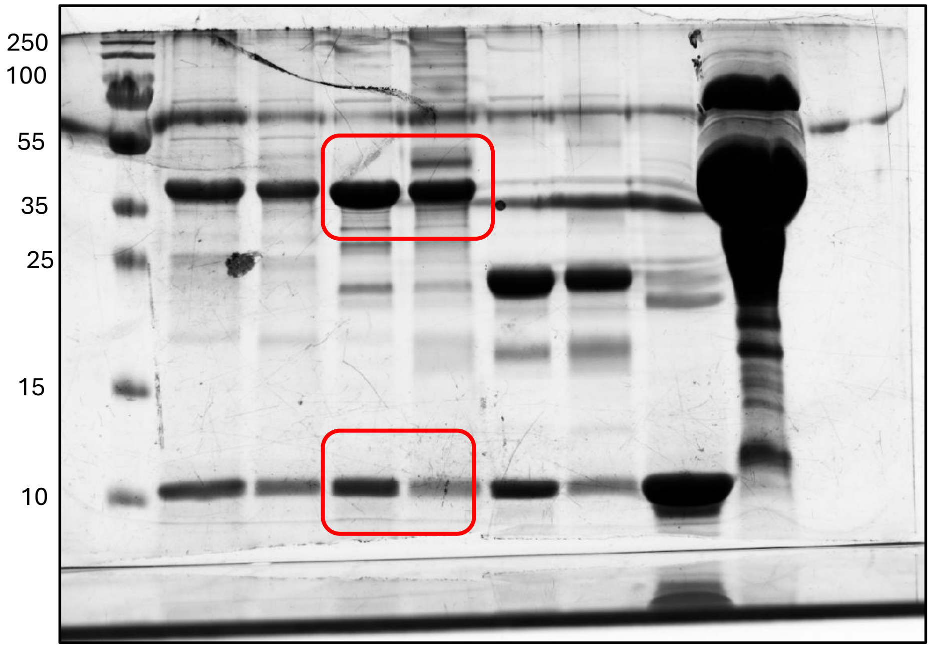

Coomassie

Supplementary Figure 9

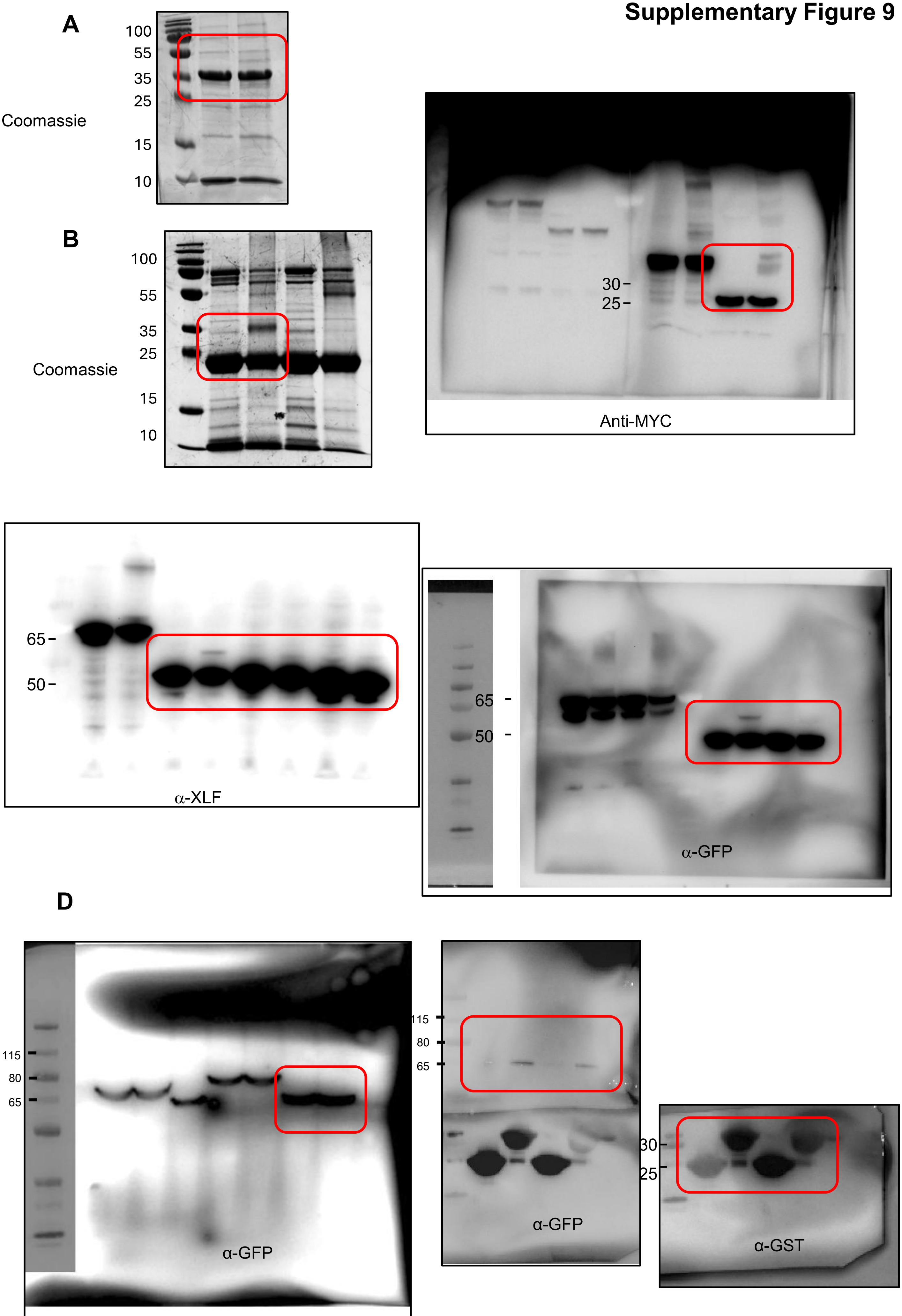

Supplementary Figure 12

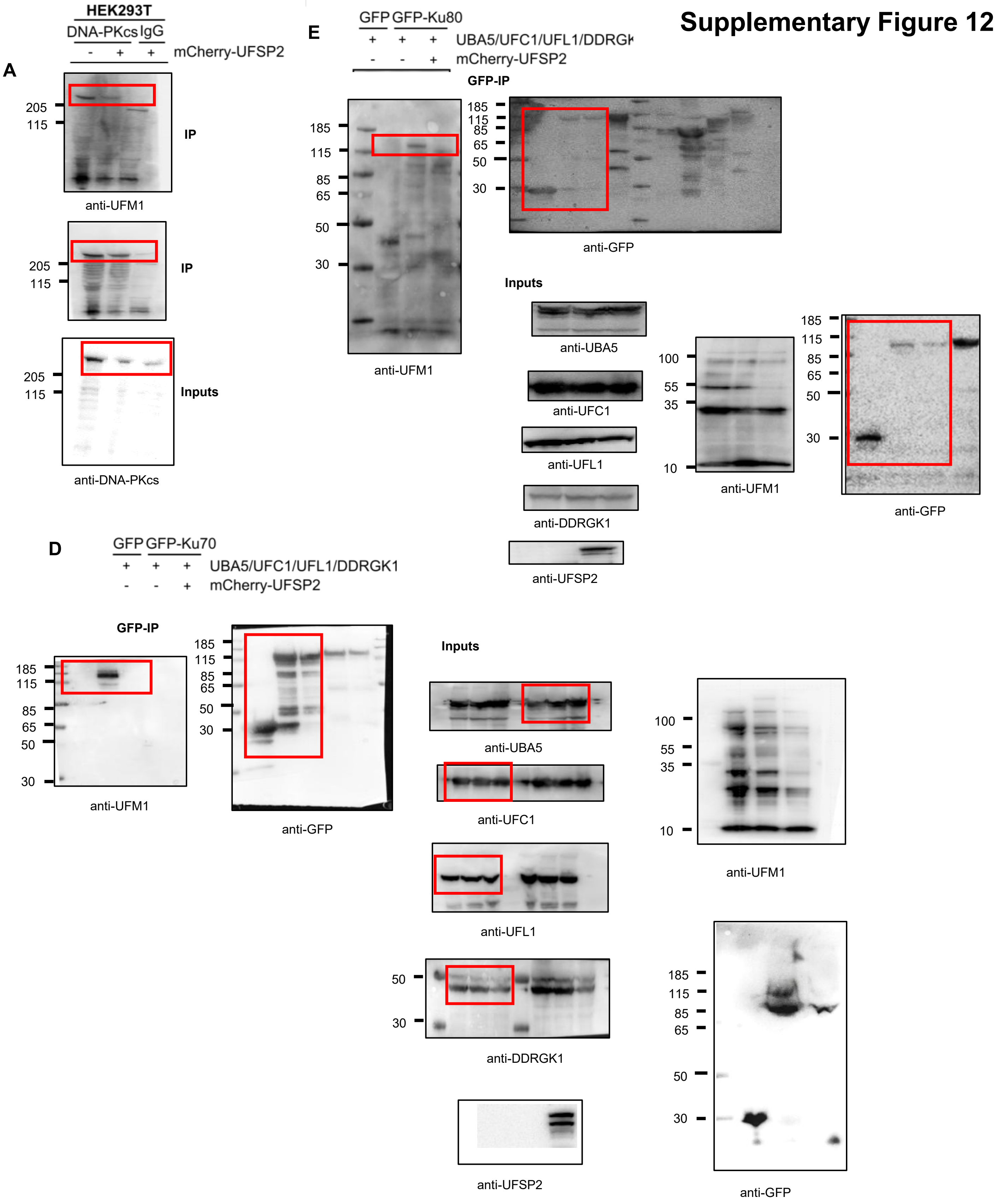

Supplementary Figure 12

B

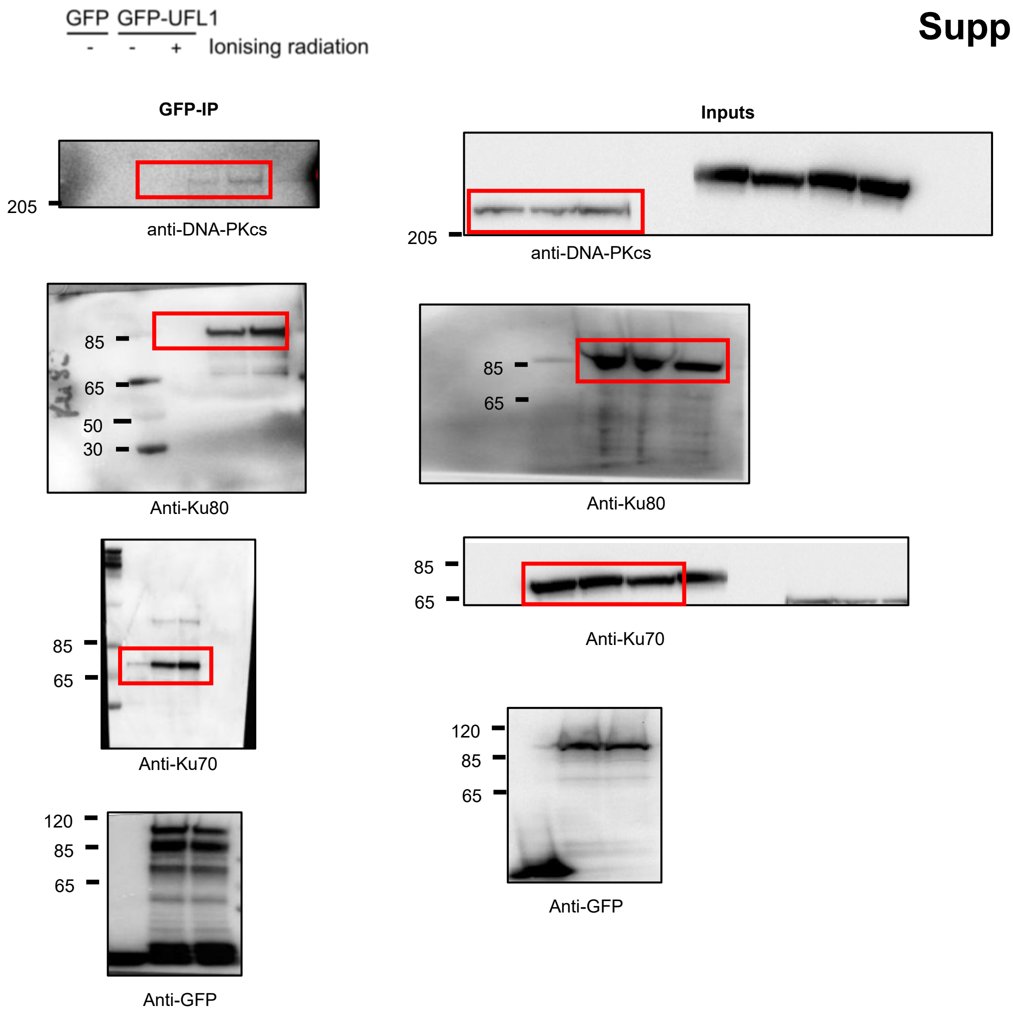

C

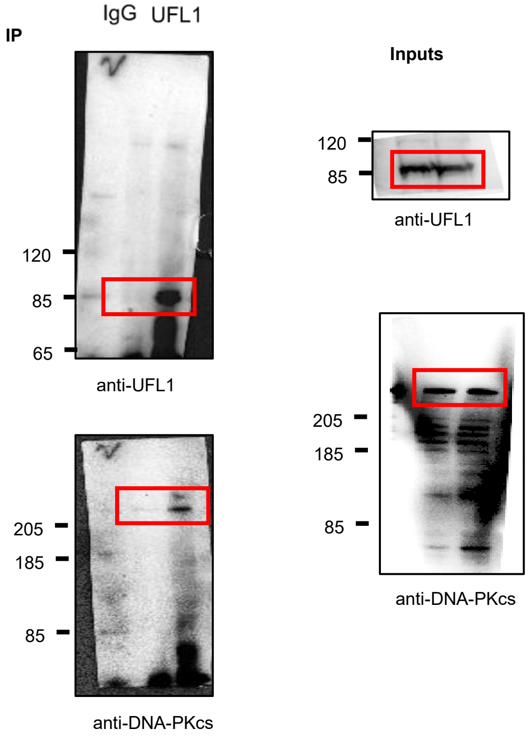

Supplement: Supplementary file 8 — Source Data [file 41467_2026_73882_MOESM8_ESM.zip › Source_data_uncropped_blots.pdf]
