# Supplementary material for: Systematic discovery of UFM1 receptors reveals a regulatory module in DNA repair directing non-homologous end-joining
Source: Nat Commun. 2026 Jun 15;17:7574. doi: 10.1038/s41467-026-73882-8 (PMC13415529; doi:10.1038/s41467-026-73882-8)
Supplement: Supplementary file 7 — Transparent Peer Review file [file 41467_2026_73882_MOESM7_ESM.pdf]

nature portfolio

Peer Review File

Systematic discovery of UFM1 receptors reveals a regulatory module in DNA repair directing non-homologous end-joining

Corresponding Author: Dr Christine Schmidt

This file contains all reviewer reports in order by version, followed by all author rebuttals in order by version.

Version 0:

Reviewer comments:

Reviewer #1

(Remarks to the Author)
The manuscript presented by Wang et al "systematic discovery of UFM1 receptors 1 reveals a regulatory module in DNA repair directing non-homologous end-joining" shows a functional role of the UFM1-UFC1-UFL1 axis in DDR including end-joining repair. The authors first performed a loss-of-function screening targeting ten UBLs and quantify HR and mutagenic EJ efficiencies. They then focused on UFM1 whose silencing affects the most the DDR. They designed an elegant UFM1 tool, a UFM1-F35-BpF probe (UFM1*) and showed that this probe can act as a robust and selective tool to capture UFM1 partners that interact transiently with it. They successfully used their probe to map by NMR three regions of XRCC4 targeted by UFM1. They also used their UFM1* probe and identified amongst UFM1 substrates DNA-PKcs, Ku70, and Ku80 by combining APEX2-based proximity labelling and SILAC approach with or without irradiation. Finally, they showed that the post-translational regulation by UFM1 enhanced assembly and/or stability of the NHEJ factors at sites of damage. This study represents an elegant approach that allowed to identify the Ubiquitin-like UFM1 substrates with a focus on NHEJ factors that should interest a large audience.

Revisions
Line 134: the authors may recall what is "mutagenic end-joining" for readers not familiar with it.
Strikingly, depletion of several UBLs with distinct DSB repair efficiency profiles, namely UFM1, ISG15, URM1, and UBL4A, also disrupted ubiquitin conjugation and/or 53BP1 IRIF formation at DNA damage sites (Fig. S1B). The authors should add error bars in Fig S1B.
Fig1D The authors should add statistical tests including between siXRCC4 and some of the siRNA
Strikingly, the top-enriched pathway was NHEJ (Fig. 3B), with all subunits of the DNA-PK complex (the catalytic subunit DNA-PKcs, Ku70, Ku80) and the core factors XRCC4 and XLF amongst the hits The variation of the enrichment among the NHEJ factors are quite different. It should be mentioned and precised
Fig 3 Legend Cell lysates were obtained from ionising radiation (IR)-treated HEK293T cells (30 min post 10 Gy). The authors may explain why they use such a high IR dose of 10Gy.
The analysis revealed five crosslinked peptides spanning three discrete regions of the XRCC4 dimer: (1) the globular head domain, (2) the coiled-coil stalk, and (3) its C-terminal intrinsically disordered region (IDR) (Fig. S5B) The residue Val47 is buried in the globular head domain. It is quite difficult to understand how the corresponding peptide may have been cross-linked.

Reviewer #2

(Remarks to the Author)
Wang et al demonstrate for the first time the importance of UFM1 to NHEJ repair. Using multiple complementary techniques, they demonstrate and map in detail UFM1 non-covalent interactions with XRCC4 and XLF, and UFM1 modification of Ku70. Importantly they reveal a plausible mechanism by which XRCC4 interacts with UFM1ylated Ku70 to stabilise NHEJ repair complexes on chromatin, and that UBA5 hypomorphic fibroblasts are defective in NHEJ repair factor assembly. A strength of this work is that multiple unbiased approaches have been used throughout which will provide an important discovery platform for other researchers interested in understudied UBLs including UFM1. This work offers an important advance in our understanding of both UBL/UFM1 biology and DSB repair mechanisms.

I am broadly in favour of publication of this research in Nature Communications. The data presented is already of high quality and depth. My suggestions are relatively minor throughout, mostly focusing on cell based readouts to strengthen the models presented of UFM1-NHEJ signalling.

Figure 1

• If the specificity of the FUBI siRNA is in doubt by the authors, the FUBI screening data should be removed from the manuscript but mentioned in the text that FUBI was included but that there was issues with specificity.

Figure 3

• Fig_3A Were any HR factors identified in this screen? This is particularly interesting considering that UFM1 scored strongly in the original screen for both HR and NHEJ.

Figure 4

• Could the multiple binding sites be an artifact of the high in vitro concentrations of the two proteins and the ability of the crosslinking to stabilise otherwise weak interactions?
• Could the authors demonstrate this region of XLF is important for UFM1 interaction in a co-ip in cells rather than by the in vitro GST pulldown assays?

Figure 5

• It is unclear why the more complex APEX-UFC1 proximity tagging approach was used here instead of denaturing pulldowns with 6xHis UFM1 (-/+ IR) as has been performed by other groups to identify UFM1 substrates (Panichnantakul et al. 2024), what are the advantages of this system over the more direct His-tagging denaturing pulldown approach?

Figure 6

• As a reciprocal experiment - do the UFM1 binding mutants (described in figure 4) of XRCC4 interact less efficiently with UFMylated Ku70?
• Do the UFM1 binding mutants of XRCC4 and UFMylation KR mutant of Ku70 show cellular NHEJ defects other than laser lines? Do deficient cells complemented with mutants of each fail to rescue IR sensitivity or NHEJ repair assays in cells?

Figure 7

• Fig_7A, these panels would benefit from a soluble and chromatin loading control rather than beta-actin.
• Unclear why the authors have switched to calicheamicin when previous assays used IR can this be explained in the text?
• The link between these UBA5 hypomorphic patients is really interesting, suggesting these syndromes may have a fairly severe DSB repair defect. To make the point stronger I suggest some DSB/IR – colony assays, or gamma-H2AX clearance kinetics, or NHEJ repair assays in WT v UBA5 hypomorph fibroblasts to further enhance this point.

Reviewer #3

(Remarks to the Author)
Wang et al. investigated the post-translational modification, specifically, UFMylation in NHEJ. First, using functional assays, they identified UFM1 as an important factor for NHEJ activity and then used crosslinking to identify the core components of NHEJ, i.e., DNA-PKcs, Ku70, XLF, and XRCC4 as the receptors of UFM1 and mapped the molecular interactions for XLF and XRCC4. Next, they identified K526 of Ku70 as the main substrate and showed that increasing global UFMylation increases the recruitment of NHEJ components to the damaged chromatin.

Overall, this manuscript provides important insights into the regulation of NHEJ by PTMs, an emerging aspect in understanding the intricate complexity of NHEJ activity.

The data presented here is solid enough to support the main claim, and I commend the authors for using very interdisciplinary approaches to characterize the transient and the complexity of UFMylation. However, this does make the manuscript a little hard to follow.

The manuscript does not provide a direct mechanism for the role of UFM1 in NHEJ activities, but the molecular basis provided here could pave the way for further mechanistic investigation.

Here, I will assess mainly the structural characterization and the functional interpretation of the manuscript.

Major remark:

- The authors should add a figure to explain the concept of UFMylation, receptors, and substrates either at the beginning to

introduce the mechanism of UFMylation or toward the end of the manuscript (to summarize the discovery of the receptors and the substrates) to help the readers who are not familiar with the mechanism of UFMylation.
- Any reason why the authors are using XRCC4(200-334) constructs but not the whole length of the disordered regions XRCC4(200-336)?
- Have the authors tried any complex prediction software like (AF or CS-constraint AF) to build the complex model between UFM1 with XLF and XRCC4? Does it agree well with the CSP/intensity loss?
- The authors should indicate that the region in the coiled-coil stalk of XRCC4 that interacts with UFM1 also is the region that interacts with the BRCTs of LIG4. Given that 2/3 of the UFM1 interaction sites in XRCC4 compete with XLF and LIG4, which are important for the recruitment of XRCC4 to DSBs, I am quite surprised that UFMylation promotes XRCC4 recruitment to DSBs, not otherwise. Could the authors come up with any explanation/hypothesis (at least in the discussion part)?

Minor remark:
- Fig. S11 is very low resolution.
- The authors should indicate the protein concentrations used in each NMR titration experiment.
- Describe in detail the methods in calculating the Z-score of CSP.

Version 1:

Reviewer comments:

Reviewer #1

(Remarks to the Author)
The reviewer appreciates the corrections made by the authors. They correspond to the points raised.

Reviewer #2

(Remarks to the Author)
All of my comments have been fully answered and I am happy to recommend publication.

Reviewer #3

(Remarks to the Author)
The authors have satisfactorily addressed the concerns with a detailed response, and I now support publication of the manuscript in its current form.

Open Access This Peer Review File is licensed under a Creative Commons Attribution 4.0 International License, which permits use, sharing, adaptation, distribution and reproduction in any medium or format, as long as you give appropriate credit to the original author(s) and the source, provide a link to the Creative Commons license, and indicate if changes were made.
In cases where reviewers are anonymous, credit should be given to 'Anonymous Referee' and the source.
The images or other third party material in this Peer Review File are included in the article’s Creative Commons license,

unless indicated otherwise in a credit line to the material. If material is not included in the article's Creative Commons license and your intended use is not permitted by statutory regulation or exceeds the permitted use, you will need to obtain permission directly from the copyright holder.
To view a copy of this license, visit https://creativecommons.org/licenses/by/4.0/

POINT-BY-POINT RESPONSE TO REFEREES' COMMENTS

We thank the Editor and the Reviewers for their careful assessment of our manuscript and for their constructive and insightful comments. We have revised the manuscript accordingly and performed additional experiments that further strengthen the mechanistic and functional conclusions of the study. We believe these revisions have improved the clarity of the manuscript and reinforced the evidence supporting a UFM1-dependent signalling axis in non-homologous end-joining. Our point-by-point responses to the Reviewers' comments are provided below in blue. Changes in the manuscript are highlighted in yellow in the revised version.

Reviewer #1:

The manuscript presented by Wang et al "systematic discovery of UFM1 receptors 1 reveals a regulatory module in DNA repair directing non-homologous end-joining" shows a functional role of the UFM1-UFC1-UFL1 axis in DDR including end-joining repair. The authors first performed a loss-of-function screening targeting ten UBLs and quantify HR and mutagenic EJ efficiencies. They then focused on UFM1 whose silencing affects the most the DDR. They designed an elegant UFM1 tool, a UFM1-F35-BpF probe (UFM1*) and showed that this probe can act as a robust and selective tool to capture UFM1 partners that interact transiently with it. They successfully used their probe to map by NMR three regions of XRCC4 targeted by UFM1. They also used their UFM1* probe and identified amongst UFM1 substrates DNA-PKcs, Ku70, and Ku80 by combining APEX2-based proximity labelling and SILAC approach with or without irradiation. Finally, they showed that the post-translational regulation by UFM1 enhanced assembly and/or stability of the NHEJ factors at sites of damage. This study represents an elegant approach that allowed to identify the Ubiquitin-like UFM1 substrates with a focus on NHEJ factors that should interest a large audience.

We thank the Reviewer for their positive and encouraging comments.

Revisions

Line 134: the authors may recall what is "mutagenic end-joining" for readers not familiar with it.

We thank the Reviewer for this comment. We have added additional context to clarify the meaning of mutagenic end-joining. The revised section now states: **"The screen was carried out using a two-pronged approach. Module 1 employed the traffic-light reporter (TLR) system to quantify DNA repair efficiencies by HR and erroneous NHEJ through mutagenic end-joining (mutEJ)[1,2] (Fig. 1A)."** (page 3 of the revised manuscript).

Strikingly, depletion of several UBLs with distinct DSB repair efficiency profiles, namely UFM1, ISG15, URM1, and UBL4A, also disrupted ubiquitin conjugation and/or 53BP1 IRIF formation at DNA damage sites (Fig. S1B). The authors should add error bars in Fig S1B.

We thank the Reviewer for noting this. We have included error bars in Figure S1B as suggested.

Fig1D The authors should add statistical tests including between siXRCC4 and some of the siRNA

Thank you to the Reviewer for highlighting this oversight. We have performed the appropriate statistical tests. Comparisons between the indicated siRNAs and siCTRL are now shown in Fig. 1D, while comparisons with siXRCC4 were also performed, which were not statistically significant. This is now indicated in the revised Figure panel and corresponding legend.

Strikingly, the top-enriched pathway was NHEJ (Fig. 3B), with all subunits of the DNA-PK complex (the catalytic subunit DNA-PKcs, Ku70, Ku80) and the core factors XRCC4 and XLF amongst the hits The variation of the enrichment among the NHEJ factors are quite different. It should be mentioned and precised.

We thank the Reviewer for highlighting this point. The varying levels of enrichment of NHEJ factors in the screen are likely due to mass spectrometry-related differences in abundance and tryptic peptide quantification, which can differ between proteins. Importantly, all validated factors were enriched to a statistically significant level across multiple biological replicates. We have clarified this point in the revised manuscript, which now states: **"While the enrichment of core NHEJ factors varied in the screen – likely due to differences in abundance or quantification of tryptic peptides – they were all significantly enriched across multiple biological replicates."** (page 6 of the revised manuscript).

Fig 3 Legend Cell lysates were obtained from ionising radiation (IR)-treated HEK293T cells (30 min post 10 Gy). The authors may explain why they use such a high IR dose of 10Gy.

We thank the Reviewer for this comment. We used 10 Gy IR because the analysis was performed at an early time point (30 min) after damage induction, which ensures strong activation of the DNA damage response and facilitates enrichment of DNA damage-regulated interactions that might otherwise be masked by a larger pool of non-damage-regulated proteins. Importantly, at this short time point the potential for confounding effects such as cell death or secondary cellular responses (e.g. cell-cycle alterations) is minimised, consistent with short-term DDR signalling experiments commonly used under these conditions in the field (e.g.[2–5]). We have clarified this rationale in the revised Figure legend (page 40 of the revised manuscript).

The analysis revealed five crosslinked peptides spanning three discrete regions of the XRCC4 dimer: (1) the globular head domain, (2) the coiled-coil stalk, and (3) its C-terminal intrinsically disordered region (IDR) (Fig. S5B) The residue Val47 is buried in the globular head domain. It is quite difficult to understand how the corresponding peptide may have been cross-linked.

We thank the Reviewer for raising this point. We agree that V47 is noteworthy in this regard. Further structural inspection revealed that while the side chain of V47 is partially buried, it retains partial surface accessibility (see Fig. 1 below, V47 highlighted in red), consistent with its ability to participate in crosslinking. To clarify this point, we have revised the manuscript (page 6), which now states: **"These studies identified significant UFM1-induced chemical shift perturbations (CSPs) and signal broadening in residues surrounding the crosslinked XRCC4 residue V47, which exhibits partial surface accessibility in the XRCC4 crystal structure (PDB 1IK9). This observation is consistent with the XL-MS crosslink and highlights a coherent surface patch, encompassing residues A56, M59, V104, and S105, as a likely interface mediating UFM1 interaction (Fig. 4A-B)."**

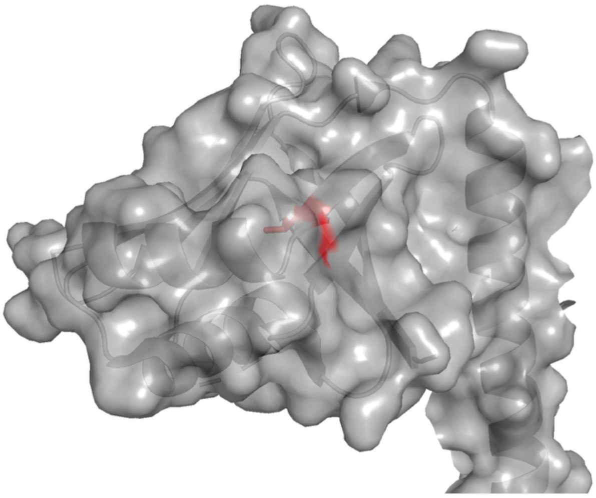

Figure 1. Surface accessibility of V47. XRCC4 surface (PDB 1IK9) of head domain, showing V47 highlighted in red.

References

1. Certo, M. T. et al. Tracking genome engineering outcome at individual DNA breakpoints. *Nat. Methods* **8**, 671–676 (2011).
2. Schmidt, C. K. et al. Systematic E2 screening reveals a UBE2D–RNF138–CtIP axis promoting DNA repair. *Nat. Cell Biol.* **17**, 1458–1470 (2015).
3. Galanty, Y., Belotserkovskaya, R., Coates, J. & Jackson, S. P. RNF4, a SUMO-targeted ubiquitin E3 ligase, promotes DNA double-strand break repair. *Genes Dev.* **26**, 1179–1195 (2012).
4. Gupta, R. et al. DNA Repair Network Analysis Reveals Shieldin as a Key Regulator of NHEJ and PARP Inhibitor Sensitivity. *Cell* **173**, 972-988.e23 (2018).
5. Galanty, Y. et al. Mammalian SUMO E3-ligases PIAS1 and PIAS4 promote responses to DNA double-strand breaks. *Nature* **462**, 935–939 (2009).

Reviewer #2

Wang et al demonstrate for the first time the importance of UFM1 to NHEJ repair. Using multiple complementary techniques, they demonstrate and map in detail UFM1 non-covalent interactions with XRCC4 and XLF, and UFM1 modification of Ku70. Importantly they reveal a plausible mechanism by which XRCC4 interacts with UFM1ylated Ku70 to stabilise NHEJ repair complexes on chromatin, and that UBA5 hypomorphic fibroblasts are defective in NHEJ repair factor assembly. A strength of this work is that multiple unbiased approaches have been used throughout which will provide an important discovery platform for other researchers interested in understudied UBLs including UFM1. This work offers an important advance in our understanding of both UBL/UFM1 biology and DSB repair mechanisms.

I am broadly in favour of publication of this research in Nature Communications. The data presented is already of high quality and depth. My suggestions are relatively minor throughout, mostly focusing on cell based readouts to strengthen the models presented of UFM1-NHEJ signalling.

We thank the Reviewer for their positive and encouraging assessment of our work and for their constructive suggestions. We are pleased that they find the study an important advance in understanding UFM1 biology and NHEJ repair. We have addressed the points raised below and performed additional analyses to further strengthen the manuscript.

Figure 1

• If the specificity of the FUBI siRNA is in doubt by the authors, the FUBI screening data should be removed from the manuscript but mentioned in the text that FUBI was included but that there was issues with specificity.

We thank the Reviewer for this suggestion and agree. We have removed the FUBI screening data from Figures S1A and S1B. A brief clarification has been retained in the manuscript text, which now states: **“Experimental analyses originally included siRNAs against FUBI but the effects observed were likely attributable to off-target activity, potentially due to sequence overlap between one of the siRNAs and UBE2I (also known as UBC9), the E2 conjugating enzyme for SUMO. Given SUMO’s well-established roles in DSB repair [5–8], FUBI was excluded from the analyses.”** (page 4 of the revised manuscript).

Figure 3

• Fig_3A Were any HR factors identified in this screen? This is particularly interesting considering that UFM1 scored strongly in the original screen for both HR and NHEJ.

We thank the Reviewer for this suggestion. Several HR-associated factors were identified in the screen. We now highlight this in the revised manuscript text, where HR- and DNA replication-associated pathways are noted as enriched (page 6 of the revised manuscript). These, together with additional DDR-related candidates-of-interest, are now annotated in the volcano plot in Figure 3A.

Figure 4

• Could the multiple binding sites be an artifact of the high in vitro concentrations of the two proteins and the ability of the crosslinking to stabilise otherwise weak interactions?

We thank the Reviewer for raising this point. As with many in vitro crosslinking approaches we cannot exclude that weak or transient interactions may be captured under the experimental conditions. To address this, we performed independent follow-up experiments that validated key interactions in cellular

systems and independently of the UFM1 probe or crosslinking reactions, including additional functional assays of NHEJ factor assembly.

We agree that it is beneficial to further acknowledge this point. We have added a sentence to the Discussion, which now states: **"How these interactions are coordinated in terms of order, local concentration and chromatin context during responses to different types of DSBs remains an interesting question for future investigation."** (page 12 of the revised manuscript).

• Could the authors demonstrate this region of XLF is important for UFM1 interaction in a co-ip in cells rather than by the in vitro GST pulldown assays?

We thank the Reviewer for this suggestion. As Figure 4 shows GST pulldown validation of UFM1-binding regions of XRCC4, which also form the primary focus of the subsequent mechanistic and functional analyses, we interpreted this comment in the context of the XRCC4 interaction regions analysed. To provide cellular validation of these interactions, we performed UFM1 co-immunoprecipitation experiments in mammalian cells as suggsted. These experiments confirmed reduced binding of UFM1-binding-deficient XRCC4 mutants compared with wildtype XRCC4 (Fig. 4F). The revised manuscript (page 7) now states:

"Co-precipitations of His-tagged UFM1 with GFP-tagged XRCC4 (wildtype and UFM1 binding-deficient mutants) further confirmed the requirement for the XRCC4 residues 180-184 in the coiled-coil region, and 256-263 in the IDR, for binding to UFM1 (Fig. 4F)."

Figure 5

• It is unclear why the more complex APEX-UFC1 proximity tagging approach was used here instead of denaturing pulldowns with 6xHis UFM1 (-/+ IR) as has been performed by other groups to identify UFM1 substrates (Panichnantakul et al. 2024), what are the advantages of this system over the more direct His-tagging denaturing pulldown approach?

The APEX2 proximity labelling strategy was used as a complementary approach to established UFMylation substrate identification methods, such as denaturing His-UFM1 pulldowns. In contrast to direct enrichment strategies, proximity labelling enables the capture of proteins proximal to the UFMylation machinery in a cellular context, including proteins in the immediate vicinity of substrate engagement under DNA damage conditions. This is particularly advantageous for identifying candidate UFMylation substrates and associated factors that may be transient or spatially restricted. Direct UFMylation of selected candidates was subsequently validated using orthogonal approaches, including confirmation of Ku70 UFMylation.

We have clarified this rationale in the revised manuscript (pages 7/8), which now states: **"To this end, we employed an inducible proximity biotinylation strategy in cells as a complementary approach to other established UFMylation substrate identification methods[9,10], including denaturing pulldown assays using His-tagged UFM1. This was to enable capture of proteins associated with the UFMylation machinery in a cellular context, including proteins engaged during substrate transfer, thereby facilitating detection of candidate substrates and factors that may be transient or spatially restricted under DNA damage conditions."**

Figure 6

• As a reciprocal experiment - do the UFM1 binding mutants (described in figure 4) of XRCC4 interact less efficiently with UFMylated Ku70?

We thank the Reviewer for this suggestion. To address this question, we performed GFP pulldown experiments following expression of GFP-tagged wild-type or mutant XRCC4 and assessed binding to endogenous Ku70. We observed reduced Ku70 binding to the XRCC4 Δ256-263 IDR mutant compared with wildtype XRCC4, whereas the XRCC4 180-5A mutant showed no marked change in Ku70 interaction (Fig. 6F).

We have incorporated these results into the revised manuscript (page 9), which now states: **"Reciprocally, GFP-XRCC4 pulldowns in *XRCC4* KO HEK293T cells further support the 256-263 IDR residues of XRCC4 as a Ku70 K526-UFM1 binding region, with reduced Ku70 co-precipitation compared with wildtype XRCC4 (Fig. 6F). By contrast, no marked changes in Ku70 binding were observed between the 180-5A XRCC4 mutant and wildtype XRCC4, suggesting that this region may interact with UFMylated substrates distinct from Ku70, consistent with a model in which multiple UFM1-dependent interactions contribute to assembly of the NHEJ complex."**

• Do the UFM1 binding mutants of XRCC4 and UFMylation KR mutant of Ku70 show cellular NHEJ defects other than laser lines? Do deficient cells complemented with mutants of each fail to rescue IR sensitivity or NHEJ repair assays in cells?

We thank the Reviewer for this question. To assess the functional consequences of disrupting UFM1-dependent interactions, we performed cellular NHEJ repair assays using the EJ7-GFP reporter system, which specifically measures end-joining without indels. These experiments were carried out in *XRCC4/XLF* double knockout HEK293T EJ7-GFP cells complemented with wildtype or mutant XRCC4 constructs. This background sensitises the assay to perturbations in NHEJ because, in the absence of XLF, loss of additional NHEJ functions becomes more readily detectable.

Using this system, we observed significantly reduced end-joining activity for all XRCC4 UFM1-binding-deficient mutants compared with wildtype XRCC4, indicating that disruption of these interactions impairs NHEJ repair in cells in a pathway-specific reporter system (Fig. 7, page 10 of the revised manuscript: **"…After normalising for transfection efficiency, WT XRCC4 restored NHEJ efficiency relative to the empty vector (EV) control, whereas all mutants showed significantly reduced repair activity…"**).

Together with the new experiments demonstrating reduced interaction between XRCC4 mutants and UFMylated Ku70 in cells (see response above), we believe the findings from these and the additional experiments performed in response to the Reviewer's suggestions substantially strengthen the molecular and functional evidence supporting the proposed UFM1-dependent signalling mechanism regulating NHEJ complex assembly. While further complementation-based interrogation of Ku70 UFMylation mutants would be informative, we note that effective and sustained loss of Ku70 in human cell systems is incompatible with sustained cell viability in standard cellular contexts, limiting the feasibility of such approaches within the scope of the current study.

Figure 7

• Fig_7A, these panels would benefit from a soluble and chromatin loading control rather than beta-actin.

We thank the Reviewer for the suggestion. To provide appropriate controls for the chromatin fractionation experiments, we have now included soluble and chromatin loading blots using tubulin and histone H3, respectively. These controls are shown for the U2OS degron samples (Fig. 8A in the revised manuscript, right panel) and the fibroblast samples (Fig. 9B in the revised manuscript, bottom left), confirming the efficiency of the chromatin fractionation in the experimental workflow.

• Unclear why the authors have switched to calicheamicin when previous assays used IR can this be explained in the text?

We thank the Reviewer for the comment. Calicheamicin was used in these experiments as an alternative DNA double-strand break-inducing agent that enables robust DNA damage signalling and chromatin recruitment of NHEJ factors, which can be reliably detected by immunoblotting in chromatin fractionation assays[11–13]. We have clarified this rationale in the revised manuscript (page 10, second paragraph).

• The link between these UBA5 hypomorphic patients is really interesting, suggesting these syndromes may have a fairly severe DSB repair defect. To make the point stronger I suggest some DSB/IR – colony assays, or gamma-H2AX clearance kinetics, or NHEJ repair assays in WT v UBA5 hypomorph fibroblasts to further enhance this point.

We thank the Reviewer for the suggestion. To directly assess DNA double-strand break repair under comparable DNA-damaging conditions, we performed neutral comet assays following induction of DSBs in patient-derived UBA5 hypomorphic fibroblasts and healthy control fibroblasts. The experiments revealed significantly slower DNA repair kinetics in the UBA5 hypomorph cells compared with repair-proficient control fibroblasts, consistent with impaired DSB repair in these syndromes. The results are presented in Figure 9C and described in the revised manuscript (page 11).

References

5. Galanty, Y. et al. Mammalian SUMO E3-ligases PIAS1 and PIAS4 promote responses to DNA double-strand breaks. *Nature* **462**, 935–939 (2009).
6. Morris, J. R. et al. The SUMO modification pathway is involved in the BRCA1 response to genotoxic stress. *Nature* **462**, 886–890 (2009).
7. Morris, J. R. & Garvin, A. J. SUMO in the DNA Double-Stranded Break Response: Similarities, Differences, and Cooperation with Ubiquitin. *J. Mol. Biol.* **429**, 3376–3387 (2017).
8. Garvin, A. J. et al. SUMO4 promotes SUMO deconjugation required for DNA double-strand-break repair. *Mol. Cell* **85**, 877-893.e9 (2025).
9. Panichnantakul, P. et al. Protein UFMylation regulates early events during ribosomal DNA-damage response. *Cell Rep.* **43**, 114738 (2024).
10. Blazev, R. et al. Site-specific quantification of the in vivo UFMylome reveals myosin modification in ALS. *Cell Reports Methods* **5**, 101048 (2025).
11. Britton, S. et al. DNA damage triggers SAF-A and RNA biogenesis factors exclusion from chromatin coupled to R-loops removal. *Nucleic Acids Res.* **42**, 9047–9062 (2014).
12. Elmroth, K. Cleavage of cellular DNA by calicheamicin γ1. *DNA Repair* **2**, 363–374 (2003).
13. Bossaert, M. et al. Identification of the main barriers to Ku accumulation in chromatin. *Cell Rep.* **43**, 114538 (2024).

Reviewer #3

Wang et al. investigated the post-translational modification, specifically, UFMylation in NHEJ. First, using functional assays, they identified UFM1 as an important factor for NHEJ activity and then used crosslinking to identify the core components of NHEJ, i.e., DNA-PKcs, Ku70, XLF, and XRCC4 as the receptors of UFM1 and mapped the molecular interactions for XLF and XRCC4. Next, they identified K526 of Ku70 as the main substrate and showed that increasing global UFMylation increases the recruitment of NHEJ components to the damaged chromatin.

Overall, this manuscript provides important insights into the regulation of NHEJ by PTMs, an emerging aspect in understanding the intricate complexity of NHEJ activity.

The data presented here is solid enough to support the main claim, and I commend the authors for using very interdisciplinary approaches to characterize the transient and the complexity of UFMylation. However, this does make the manuscript a little hard to follow.

The manuscript does not provide a direct mechanism for the role of UFM1 in NHEJ activities, but the molecular basis provided here could pave the way for further mechanistic investigation.

Here, I will assess mainly the structural characterization and the functional interpretation of the manuscript.

We thank the Reviewer for their positive and constructive assessment of our work and for highlighting the importance of understanding posttranslational regulation of NHEJ. We appreciate the Reviewer's recognition of the interdisciplinary approaches we used in the study. We have addressed the specific points raised below and have revised the manuscript to improve clarity.

Major remark:

- The authors should add a figure to explain the concept of UFMylation, receptors, and substrates either at the beginning to introduce the mechanism of UFMylation or toward the end of the manuscript (to summarize the discovery of the receptors and the substrates) to help the readers who are not familiar with the mechanism of UFMylation.

We thank the Reviewer for this suggestion and agree that an Overview Figure would aid readers less familiar with the UFMylation pathway. We have added a schematic representation of the UFM1 pathway in Supplementary Fig. S4, illustrating the core enzymatic components as well as the concepts of UFM1 substrates and receptors involved in downstream signalling. References to this schematic have been incorporated into the revised manuscript (page 4), which now states:

"Like ubiquitin and other well-characterised UBLs, UFM1 is conjugated to substrates via an enzymatic cascade, consisting of a single activating E1 enzyme (UBA5), a conjugating E2 enzyme (UFC1) and an E3 ligating complex (UFL1 in association with DDRGK1, also known as UFBP1)[14–18] (Fig. S4), with relatively few downstream UFM1 receptors currently known."

and

"Given the important role of downstream UBL receptors in determining the outcomes of UBLylation events (Fig. S4), we employed a bottom-up strategy to systematically identify UFM1 receptors."

- Any reason why the authors are using XRCC4(200-334) constructs but not the whole length of the disordered regions XRCC4(200-336)?

The XRCC4 constructs used in our study correspond to the Q13426-2 isoform, which differs from the 336 amino acid-long Q13426-1 isoform by featuring a K at position 298 instead of an NSR insertion at positions 298-300. As a result, the corresponding C-terminal fragment spans residues 200-334 rather than 200-336. This isoform was used throughout the study and corresponds to the variant originally used in the identification of XRCC4 as a factor involved in end-joining repair[19,20]. Importantly, this difference lies outside the regions of interest in our structural and interaction studies, and no functional differences between these isoforms have been reported. We agree that this distinction should be clarified and have updated the manuscript text and reagent Table S3 to explicitly indicate the XRCC4 isoform used (pages 21 and 25 of the revised manuscript).

- Have the authors tried any complex prediction software like (AF or CS-constraint AF) to build the complex model between UFM1 with XLF and XRCC4? Does it agree well with the CSP/intensity loss?

We thank the Reviewer for the suggestion. We performed AlphaFold-based complex prediction for UFM1 and XRCC4. The resulting model (see below) supports a UFM1-XRCC4 interface consistent with our experimental observations. In particular, the region surrounding UFM1 residue F35 (highlighted in blue), where the photo-crosslinkable residue was introduced, is predicted to dock against XRCC4 residues implicated by our NMR analysis, including V104 and M59 (highlighted in red) as well as S105 and A56, which showed significant CSPs or signal intensity loss in the NMR experiments (Fig. 4A of the revised manuscript; z-scores >3 shown in red and >2 in orange). The predicted interface is now integrated into the revised manuscript as Supplementary Figure S6C. The incorporated analysis can be found on page 7 of the revised manuscript, which now states:

"This observation is consistent with the XL-MS crosslink and highlights a coherent surface patch, encompassing residues A56, M59, V104, and S105, as a likely interface mediating UFM1 interaction (Fig. 4A-B). **AlphaFold3 complex modelling of XRCC4 and UFM1 further supports this region as a plausible interaction surface (Fig. S6C).**"

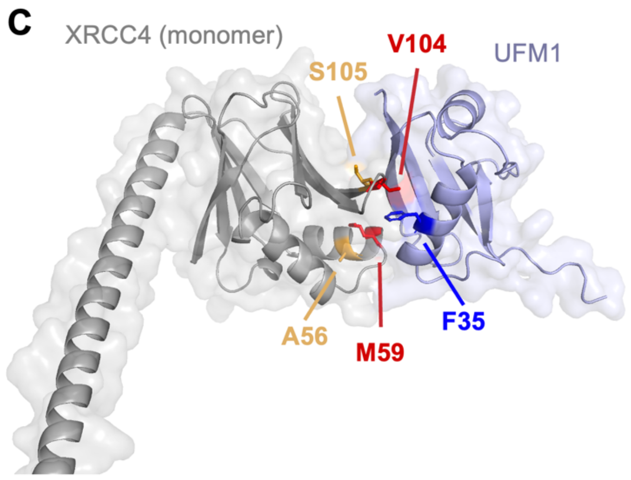

- The authors should indicate that the region in the coiled-coil stalk of XRCC4 that interacts with UFM1 also is the region that interacts with the BRCTs of LIG4. Given that 2/3 of the UFM1 interaction sites in XRCC4 compete with XLF and LIG4, which are important for the recruitment of XRCC4 to DSBs, I am quite surprised that UFMylation promotes XRCC4 recruitment to DSBs, not otherwise. Could the authors come up with any explanation/hypothesis (at least in the discussion part)?

We thank the Reviewer for this comment. We agree that the presence of multiple UFM1-interacting regions in XRCC4, some of which overlap with known NHEJ interaction surfaces, is an important point to address to clarify how these interactions may be coordinated during end-joining repair. As suggested, we have expanded the Discussion to address this point (page 12 of the revised manuscript). The revised text now reads:

"Notably, two of the identified UFM1 interaction surfaces on XRCC4 overlap with regions previously implicated in binding XLF and the LIG4 BRCT domains[21–26]. While at first glance this might suggest potential competition between these interactions, our findings indicate that UFMylation has overall promoting effects on NHEJ recruitment and efficiency. Consistent with our identification of Ku70 as a UFMylated substrate engaging XRCC4, these observations suggest that UFM1 engagement may instead regulate the ordering or dynamics of XRCC4 interactions during NHEJ complex assembly. For example, transient UFM1 binding could promote a recruitment-competent state of XRCC4 or stabilise intermediate repair assemblies at sites of damage prior to, or in coordination with, XLF and LIG4 association. In this model, UFMylation would contribute to the multivalent assembly and stabilisation of the NHEJ machinery rather than simply competing with established XRCC4 interactions.

Alternatively, within the XRCC4 head domain – UFM1 binding overlapping with the XLF interaction surface may provide an additional layer of regulatory flexibility within the NHEJ network. Given known redundancy and buffering capacity associated with XLF functions in NHEJ[27], transient UFM1 engagement could help maintain efficient recruitment of NHEJ factors under conditions where XRCC4-XLF interactions are weakened or dynamically regulated. How exactly these interactions are coordinated in terms of their temporal order, structural configuration, local concentration, and chromatin context during responses to different types of DNA double-strand breaks remains an important question for future investigation."

This addition clarifies that UFM1 binding may regulate the ordering and dynamics of XRCC4 interactions during NHEJ complex assembly, supporting efficient recruitment of repair factors without excluding established roles of XLF and LIG4 in the repair complex.

Minor remark:

- Fig. S11 is very low resolution.

We thank the Reviewer for flagging this. We have replaced the Figure with a higher-resolution version (Figure S12 in the revised manuscript).

- The authors should indicate the protein concentrations used in each NMR titration experiment.

We thank the Reviewer for highlighting this – we agree. We have included the concentrations of proteins used in the NMR titration experiments in the Figure legends of the corresponding experiments. Briefly, in Figure 4A and Supplementary Figure S6A we used 100 μM ^{15}N XRCC4 with 100 μM (1:1) and 500 μM (1:5) UFM1; in Figure 4D we used 195 μM ^{15}N XRCC4 with 195 μM UFM1 (1:1); in Supplementary Figures S9A/B and S10A/B we used 100 μM ^{15}N UFM1 (1:1) and in S9C/10C 50 μM ^{15}N UFM1 (1:1).

- Describe in detail the methods in calculating the Z-score of CSP.

Agreed. As suggested, we have included the method for calculating this in the Materials and Methods section of the revised manuscript (page 24), which reads as follows:

"Z-score of chemical shift perturbations (CSP) used the standard deviations (sigma) taken from the root mean squared differences in HSQC spectra peak positions, using the equation:

$$z = \sqrt{\left(\left(\frac{(\delta H_{free} - \delta \mathrm{H}_{bound})}{\sigma H}\right)^2 + \left(\frac{(\delta N_{free} - \delta N_{bound})}{\sigma N}\right)^2\right)}$$

where the uncertainties sH = 0.005 ppm and sN = 0.03 ppm for both XLF and XRCC4 experiments and "bound" spectra was the 1:1 mix of UFM1 with the binding protein."

References

14. Komatsu, M., Inada, T. & Noda, N. N. The UFM1 system: Working principles, cellular functions, and pathophysiology. *Mol. Cell* **84**, 156–169 (2024).
15. Tatsumi, K. et al. A Novel Type of E3 Ligase for the Ufm1 Conjugation System. *Journal of Biological Chemistry* **285**, 5417–5427 (2010).
16. Cai, Y. et al. UFBP1, a Key Component of the Ufm1 Conjugation System, Is Essential for Ufmylation-Mediated Regulation of Erythroid Development. *PLoS Genet.* **11**, e1005643 (2015).
17. Peter, J. J. et al. A non-canonical scaffold-type E3 ligase complex mediates protein UFMylation. *EMBO J.* **41**, e111015 (2022).
18. Ishimura, R. et al. The UFM1 system regulates ER-phagy through the ufmylation of CYB5R3. *Nat. Commun.* **13**, 7857 (2022).
19. Critchlow, S. E., Bowater, R. P. & Jackson, S. P. Mammalian DNA double-strand break repair protein XRCC4 interacts with DNA ligase IV. *Current Biology* **7**, 588–598 (1997).
20. Li, Z. et al. The XRCC4 gene encodes a novel protein involved in DNA double-strand break repair and V(D)J recombination. *Cell* **83**, 1079–1089 (1995).
21. Sibanda, B. L. et al. Crystal structure of an Xrcc4-DNA ligase IV complex. Nat. Struct. Biol. 8, 1015–1019 (2001).
22. Hammel, M., Yu, Y., Fang, S., Lees-Miller, S. P. & Tainer, J. A. XLF Regulates Filament Architecture of the XRCC4·Ligase IV Complex. *Structure* **18**, 1431–1442 (2010).
23. Wu, Q. et al. Non-homologous end-joining partners in a helical dance: structural studies of XLF–XRCC4 interactions. *Biochem. Soc. Trans.* **39**, 1387–1392 (2011).
24. Andres, S. N. et al. A human XRCC4–XLF complex bridges DNA. *Nucleic Acids Res.* **40**, 1868–1878 (2012).
25. Ropars, V. et al. Structural characterization of filaments formed by human Xrcc4–Cernunnos/XLF complex involved in nonhomologous DNA end-joining. *Proceedings of the National Academy of Sciences* **108**, 12663–12668 (2011).
26. Hammel, M. et al. XRCC4 Protein Interactions with XRCC4-like Factor (XLF) Create an Extended Grooved Scaffold for DNA Ligation and Double Strand Break Repair. *Journal of Biological Chemistry* **286**, 32638–32650 (2011).
27. Stinson, B. M. & Loparo, J. J. Repair of DNA Double-Strand Breaks by the Nonhomologous End Joining Pathway. *Annu. Rev. Biochem.* **90**, 137–164 (2021).
